# Recent Advances in Nanocomposite Hydrogel Sensors for Healthcare Applications: Fabrication Techniques, Multifunctionality and Challenges

**DOI:** 10.3390/nano16191211

**Published:** 2026-09-24

**Authors:** Md M. Hossain, Tarikul Islam, Mehedi H. Chaion, Mahbubay Rabbani, Md. A. Islam, Mukitur Rhaman, Abu S. Rafi, Md. A. Shahid, Subrata C. Das

**Affiliations:** 1 Department of Wet Processing Engineering, Bangladesh University of Textiles, Dhaka 1208, Bangladesh; 2 Department of Textile Engineering, Jashore University of Science and Technology, Jashore 7408, Bangladesh; 3 Department of Textiles, Merchandising, and Interiors, University of Georgia, Athens, GA 30602, USA; 4 Department of Textile Engineering, Dhaka University of Engineering and Technology, Gazipur 1707, Bangladesh; 5 Department of Textile Engineering, Northern University Bangladesh, Dhaka 1230, Bangladesh; 6 Department of Textile Engineering, Khulna University of Engineering and Technology, Khulna 9203, Bangladesh; 7 Department of Textile Engineering, University of Scholars, Dhaka 1213, Bangladesh; 8 Advanced and Sustainable Engineering Materials Laboratory (ASEMlab), Department of Manufacturing and Civil Engineering, Norwegian University of Science and Technology, 2815 Gjøvik, Norway

**Keywords:** nanocomposites (NCs), nanomaterials (NMs), MXene, polymer composites, hydrogels (HGs), wearable sensors, biocompatibility, electrical conductivity, healthcare applications, self-healing

## Abstract

Nanocomposite (NC) hydrogel (HG) sensors have emerged as a transformative innovation in wearable healthcare monitoring systems. By incorporating carbon-based, metal-based, and polymer-based NCs, these sensors offer exceptional properties such as biocompatibility, flexibility, high water content, and stretchability—making them highly suitable for continuous, real-time physiological monitoring. Advanced fabrication techniques, including in situ polymerization, conformal coating, and dynamic crosslinking, significantly enhance their functional performance and scalability for practical deployment. The mechanical robustness and electrical responsiveness of these NC HGs, coupled with self-healing capabilities, strong adhesion to skin, and resistance to environmental stressors, ensure their reliability and durability in diverse healthcare applications. These HG sensors have shown great promise in monitoring vital signs, managing chronic conditions, supporting rehabilitation, enabling real-time glucose monitoring, and facilitating wound healing. Despite their considerable potential, several challenges remain—particularly in technical optimization, user comfort and integration, and regulatory approval—that must be addressed to enable their widespread adoption. This review highlights recent innovations, material properties, and healthcare applications of NC HG sensors and discusses the critical challenges that must be overcome to fully realize their impact as wearable textile materials in modern healthcare systems.

## 1. Introduction

The rapid advancement of wearable sensor technology has significantly simplified healthcare monitoring systems, enabling more precise and real-time tracking of physiological parameters and enhancing patient care [1,2]. A wide range of sensors—such as anti-freezing hydrogels (HGs), dual-network HG sensors, MXene nanocomposite (NC) organo-HG sensors, and healable epidermal sensors—have been explored for healthcare applications. Among these, nanocomposite hydrogels (NC HGs) stand out due to their unique combination of biocompatibility, flexibility, and high water content, making them ideal for continuous health monitoring and close contact with the human body [3]. These HGs are typically synthesized by incorporating conductive fillers such as carbon nanotubes (CNTs) and graphene, which significantly enhance their electrical conductivity [4]. Their high conductivity and conformal adhesion to the skin allow for the detection of subtle physiological signals, such as pulse and respiration—with remarkable precision [5,6].

The exceptional mechanical properties of NC HGs—including high stretchability, robustness, sensitivity, and biocompatibility—make them versatile tools for applications such as motion tracking and physiological monitoring [7,8,9]. Compared to traditional rigid sensors, HG-based NC sensors offer numerous advantages: they are lightweight, flexible, and self-healing and exhibit strong adhesion to various surfaces, thereby improving both technical performance and user comfort [10,11,12]. Rigid sensors often hinder movement and cause discomfort, whereas HG-based alternatives reduce the risk of skin irritation and adverse reactions, making them more suitable for long-term wear [13]. The integration of ionic conductors further enhances the sensing range and conductivity of the HGs, which is particularly beneficial for chronic disease management [14]. Additionally, technologies such as Bluetooth Low Energy (BLE) sensors, wearable inertial sensors, and microsystem sensors provide real-time feedback, facilitating timely medical interventions and empowering individuals to take control of their health [15,16,17]. Multichannel wireless sensing systems have been developed to monitor a wide array of physiological signals—including heart rate, electrocardiogram (ECG), electromyography (EMG), respiratory activity, glucose levels, and other biomarkers—enabling comprehensive health assessments through wearable textiles. These systems, combined with advanced signal processing, allow for continuous, real-time data collection and health evaluation [18,19,20].

HG biosensors represent a paradigm-shifting platform in healthcare by successfully reconciling the mechanical and chemical discrepancies between rigid, conventional electronics and delicate human tissue. These materials excel due to their inherent three-dimensional (3D) porous architecture, high water retention, and synergistic ionic-electronic conduction. This unique structure accelerates mass transport and maximizes functional interface areas, which dramatically enhances real-time detection sensitivity [21]. Furthermore, HGs provide high cell viability, minimal cytotoxicity, and rapid response times to acute physiological fluctuations. These properties allow for precise, non-invasive analysis of biofluids (like sweat and interstitial fluid), as well as complex internal monitoring via cardiac and neural implants without causing severe immune rejection. By merging adaptive chemistry with mechanical flexibility, HG sensors have transitioned from mere protective coatings to essential tools for autonomous, precision medicine [21,22]. Their inherent properties—such as tunable cross-linking, biocompatibility, and rapid molecular diffusion—dictate how effectively they perform across diverse biomedical applications [21].

HGs possess an ultra-low Young’s modulus matching human skin, allowing them to form conformal, seamless interfaces without motion artifacts. They are widely applied as epidermal electronic patches to continuously monitor physical vital signs like heart rate, respiration, and joint movement [23,24]. The intrinsic 3D porous network of HGs permits rapid diffusion of small molecules. This makes them ideal for sampling non-invasive biofluids (such as sweat, tears, and interstitial fluid) to selectively capture target biomarkers like glucose, lactate, cortisol, and tumor-specific proteins [21,22]. Traditional electronic implants often trigger a foreign body response due to a severe mechanical mismatch with soft tissue. HGs mitigate this risk by structurally mimicking the extracellular matrix. They act as soft, non-retractive coatings on neural electrodes, cardiac patches, or deep-tissue strain gauges, safely integrating with internal organs [21,22]. Beyond discrete sensing, HGs function as multi-functional therapeutic platforms. In smart wound dressings, they can simultaneously monitor localized pH or temperature fluctuations (indicative of bacterial infection) while managing moisture and releasing on-demand therapeutics [23,25].

The physical configuration of an HG directly determines its suitability for distinct transduction mechanisms and healthcare applications. Electrically, hydrogels can leverage dissolved mobile ions for metal-free ionic sensing to capture electrophysiological signals (ECG/EMG) [24], embed conductive fillers for piezoresistive motion tracking via shifted percolation thresholds, or act as compliant electrodes for rapid-recovery capacitive tactile pressure arrays [24,26]. Chemically, their 3D porous networks facilitate the mass transport of target analytes required for high-sensitivity electrochemical biosensing of biomarkers like glucose [21], while stimuli-responsive swelling enables optical or colorimetric sensing via physical shifts in light transmission [26]. Ultimately, the biomedical utility of these transduction methods relies on three architectural pillars: viscoelasticity and mechanical robustness derived from dynamic networks to withstand physiological stresses [27]; a high swelling ratio to eliminate signal latency during molecular diffusion [22]; and tailored surface chemistries providing antifouling capabilities to resist immune rejection in long-term implantable biointerfaces.

Recent research has increasingly focused on enhancing the adhesion, mechanical strength, and electrical performance of HG-based sensors [13]. For instance, Thirumalai et al. [28] reviewed the state-of-the-art in conductive polymer-based HGs for wearable electrochemical biosensors, emphasizing the role of nanomaterials (NMs) in improving functionality and addressing key challenges such as strain sensing range, signal variability, dehydration, and fabrication complexity. Shi et al. [29] introduced a multifunctional PAM/BA-Ag@PDA HG synthesized via in situ polymerization, demonstrating high sensitivity, rapid response, and excellent biocompatibility, features well-suited for future wearable healthcare applications. Liu et al. [30] developed a high-ion-conducting polyvinyl alcohol (PVA) NC HG incorporating MXene, PVA, poly(styrene sulfonate) (PSS), and glycerol, which exhibited superior sensitivity and mechanical-electronic conversion across a wide temperature range. Complementing these developments, recent reviews have highlighted the growing significance of NC HG systems in biomedical applications. Moghaddasi et al. [31] synthesized findings from 125 studies, highlighting the predominance of carbon-based NMs, metals, conductive polymers, and hybrid systems in neural tissue engineering, along with emerging biomedical applications. Wahid et al. [32] provided a comprehensive overview of multifunctional NC HGs, demonstrating how the incorporation of diverse NMs—including carbon-based, silica, polymeric, and metal nanoparticles (NPs)—enhances mechanical, physicochemical, and biological properties through synergistic effects. In a recent study, Lin et al. [33] focused on NM-based composite HG sensors for human–machine interactions (HMIs), detailing how the integration of carbon NMs, metals, and MXenes improves flexibility, conductivity, and multifunctionality, with applications in wearable electronics, virtual (VR)/augmented reality (AR) interfaces, and robotic control. Alshangiti et al. [34] reviewed macromolecular carbohydrate-based NC HG biomaterials, emphasizing the role of natural biopolymers such as cellulose, chitosan, alginate, hyaluronic acid, and carrageenan in improving mechanical performance and controlled drug release, while maintaining biocompatibility, with applications in tissue engineering, drug delivery, wound healing, and biosensing. In this context, the present review aims to provide a focused synthesis of recent advancements in NC HG sensors specifically for wearable healthcare applications. It examines material innovations, functional properties, and integration strategies within wearable technologies, while addressing sustainability and current challenges. By concentrating exclusively on healthcare-related studies, this review highlights the transformative potential of NC HG sensors in enabling real-time diagnostics, personalized care, and continuous health monitoring.

## 2. Nanocomposite (NC) Hydrogels (HGs)

### 2.1. Carbon-Based NC HGs

Carbon-based NC HGs possess 3D hierarchical structures capable of retaining substantial amounts of water, which contributes to their unique functional properties and broad applicability [35]. Their structural tunability and ability to incorporate diverse NMs make them superior to many conventional materials (see Figure 1a). The synthesis of carbon-based NC HGs typically involves integrating carbonaceous materials—such as graphene, CNTs, and biomass-derived carbon—into HG matrices [36]. This synthesis can be broadly classified into 3 types: (i) graphene-based, (ii) polymer-based, and (iii) biomass-derived HGs [37]. Each type offers distinct advantages tailored to specific applications.

Graphene-based HGs, derived from 2D graphene or graphene oxide (GO), exhibit outstanding mechanical strength and electrical conductivity. Functionalization with various groups and NMs further enhances their versatility [38]. Polymer-based HGs provide excellent flexibility and tunability due to their crosslinked polymer networks [39]. Biomass-derived HGs, sourced from renewable materials, offer a sustainable and environmentally friendly alternative for green chemistry applications.

Carbon-based NC HGs have attracted significant attention in energy storage, environmental sensing, and wearable electronics. Their intrinsic conductivity and porous architecture enable efficient electron transport and rapid ion diffusion, resulting in superior electrochemical performance [40]. The ability to incorporate sustainable, non-toxic, and cost-effective functional moieties during synthesis further enhances their appeal for next-generation technologies. These HGs are ideal for energy conversion systems, such as supercapacitors, lithium-ion batteries, fuel cells, and water electrolyzes, where they improve performance, stability, and efficiency. In wearable devices, their biocompatibility and tunable mechanical properties support the development of flexible, non-invasive sensors for real-time health monitoring. Additionally, their conductivity facilitates human–computer interaction, enabling sensitive touch interfaces for advanced virtual and augmented reality systems [41,42,43,44,45].

**Figure 1:**
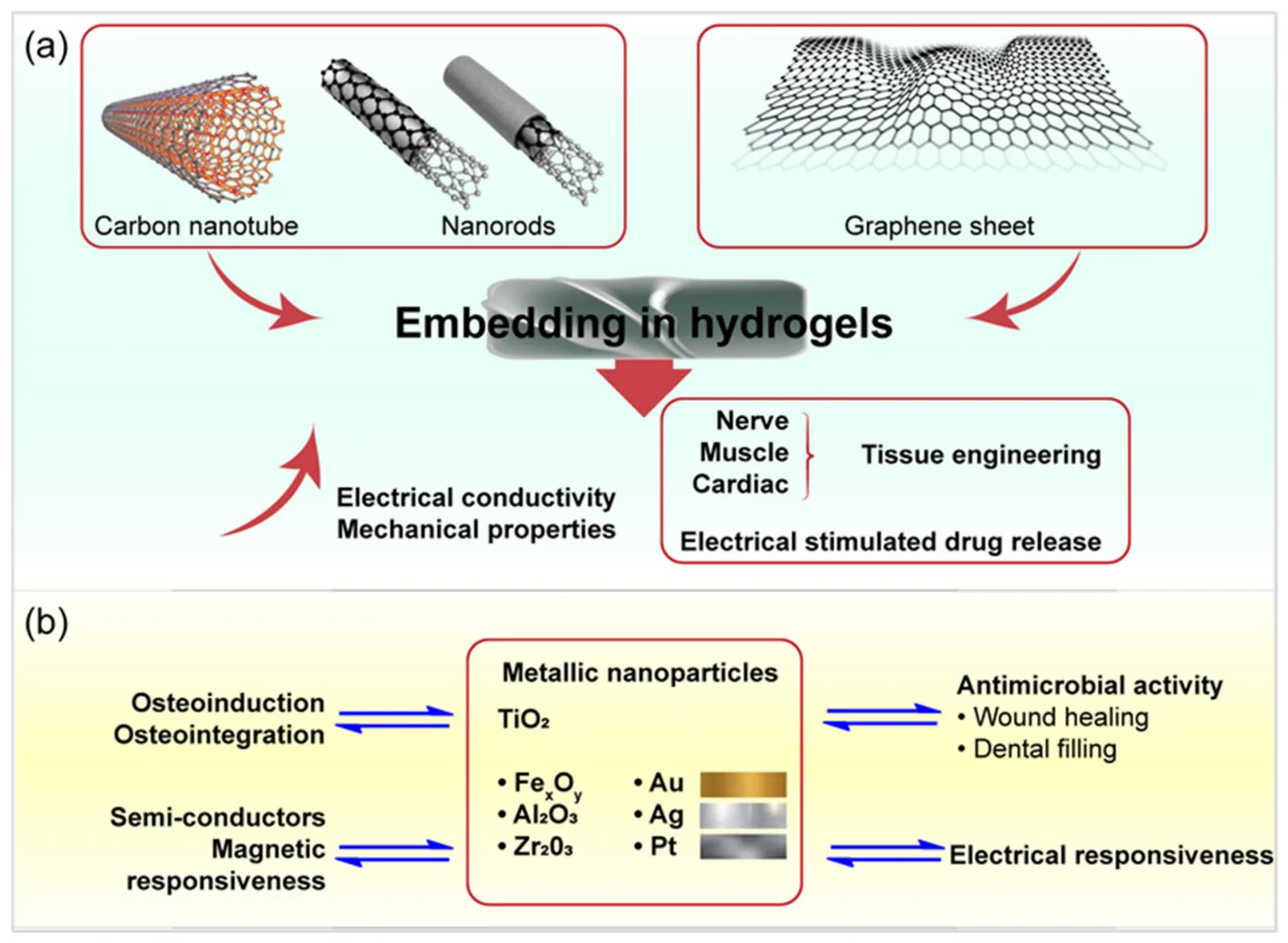
Schematic illustration of nanocomposite (NC) hydrogel (HG) architectures: (**a**) carbon-based NC HGs incorporating conductive carbon nanomaterials (NMs) [46], and (**b**) metal-based NC HGs [46].

### 2.2. Metal-Based NC HGs

Metal-based NC HGs combine the functional advantages of metal ions and metal/metal oxide NPs with the versatility of HG matrices, enabling a wide range of advanced applications. Incorporating metals such as silver, copper, and zinc enhances properties like antibacterial activity, conductivity, catalytic efficiency, and mechanical strength, making these HGs ideal for biomedical, environmental, and electronic technologies [47,48,49]. The synthesis of metal-based NC HGs involves techniques such as in situ reduction of metal ions, physical entrapment of pre-synthesized NPs, and surface functionalization to improve compatibility with the HG network [50]. Their performance can be tuned by adjusting parameters like metal concentration, NP size, dispersion, and polymer composition [51]. Understanding the interactions between metal components and the HG matrix is essential for optimizing functionality, stability and biocompatibility.

These HGs support diverse applications: in biomedicine, they facilitate tissue engineering, drug delivery, wound healing, and biosensing; in water treatment, they remove heavy metals, pathogens, and organic pollutants through high adsorption and redox activity; and in electronics, their conductivity and flexibility enable wearable sensors and soft flexible electrodes. Additionally, their catalytic properties make them effective platforms for chemical reactions [52,53,54,55,56,57].

### 2.3. Polymer-Based NC HGs

Polymer-based NC HGs are hybrid materials formed by incorporating NPs into polymer networks, significantly enhancing the physical, chemical and electrical properties of conventional HGs [57] (see Figure 2). The interaction between polymer chains and carbon-based NPs enables the design of advanced nano-reinforcements, contributing to the development of next-generation biomaterials [57,58]. Due to their porous and highly hydrated 3D structure—capable of retaining up to 90–99% water—these HGs closely mimic native tissue environments, making them highly suitable for biomedical applications [57,59]. Their dynamic interaction with cells and multifunctional capabilities support a wide range of uses in biomedicine, including cancer therapy, regenerative medicine, drug delivery, biosensing, and bio-actuation. These sensors demonstrate immense potential in both biomedical and technological fields, offering a versatile foundation for the development of smart, responsive, and biocompatible systems [59,60].

Besides conventional synthetic polymer networks, ionically and enzymatically crosslinked biopolymers are emerging as promising sustainable alternatives for ionically conductive gels and wearable electronics. Biopolymers such as alginate, chitosan, gelatin, and casein are obtained from renewable resources and offer advantages including biocompatibility, mild processing conditions, and potential biodegradability [61,62]. Their polymer networks can be formed through ionic or enzymatic crosslinking, providing an alternative to conventional chemical crosslinking methods. In particular, enzymatic crosslinking enables the formation of stable biopolymer networks under mild conditions while maintaining the functional properties of the polymer [63,64]. Recent studies have demonstrated the development of fully bio-based gelatin organo-HGs through enzymatic crosslinking for soft strain and temperature sensing applications, highlighting the potential of this approach for sustainable wearable electronics [65]. Similarly, the crosslinking strategy of chitosan-based HGs plays an important role in controlling their network structure and rheological properties, which can directly influence their mechanical performance and potential applications [66]. Therefore, the use of ionically and enzymatically crosslinked biopolymers provides a promising strategy for developing sustainable, biocompatible, and potentially biodegradable conductive gel systems for next-generation wearable electronics.

The fundamental distinction among the three primary classes of NC HGs lies in the nature of their reinforcing materials, which determine their core functional properties and application domains (see Table 1). Carbon-based NC HGs, incorporating NMs such as graphene and CNTs, are engineered for superior electrical conductivity and mechanical robustness. These characteristics make them particularly suitable for applications in wearable electronics, flexible sensors, and advanced human–machine interface technologies. In contrast, metal-based NC HGs, which utilize metal NPs such as silver or copper, are distinguished by their enhanced antibacterial and catalytic properties. These HGs are predominantly applied in biomedical fields, such as wound healing and biosensing, as well as in environmental technologies, including water purification. Polymer-based NC HGs are primarily defined by the structural characteristics of their polymer matrices, which typically exhibit highly hydrated and porous architectures that mimic native biological tissues. This biomimetic design enables their effective use in a wide range of biomedical applications, including regenerative medicine, targeted drug delivery, and cancer therapy.

**Figure 2:**
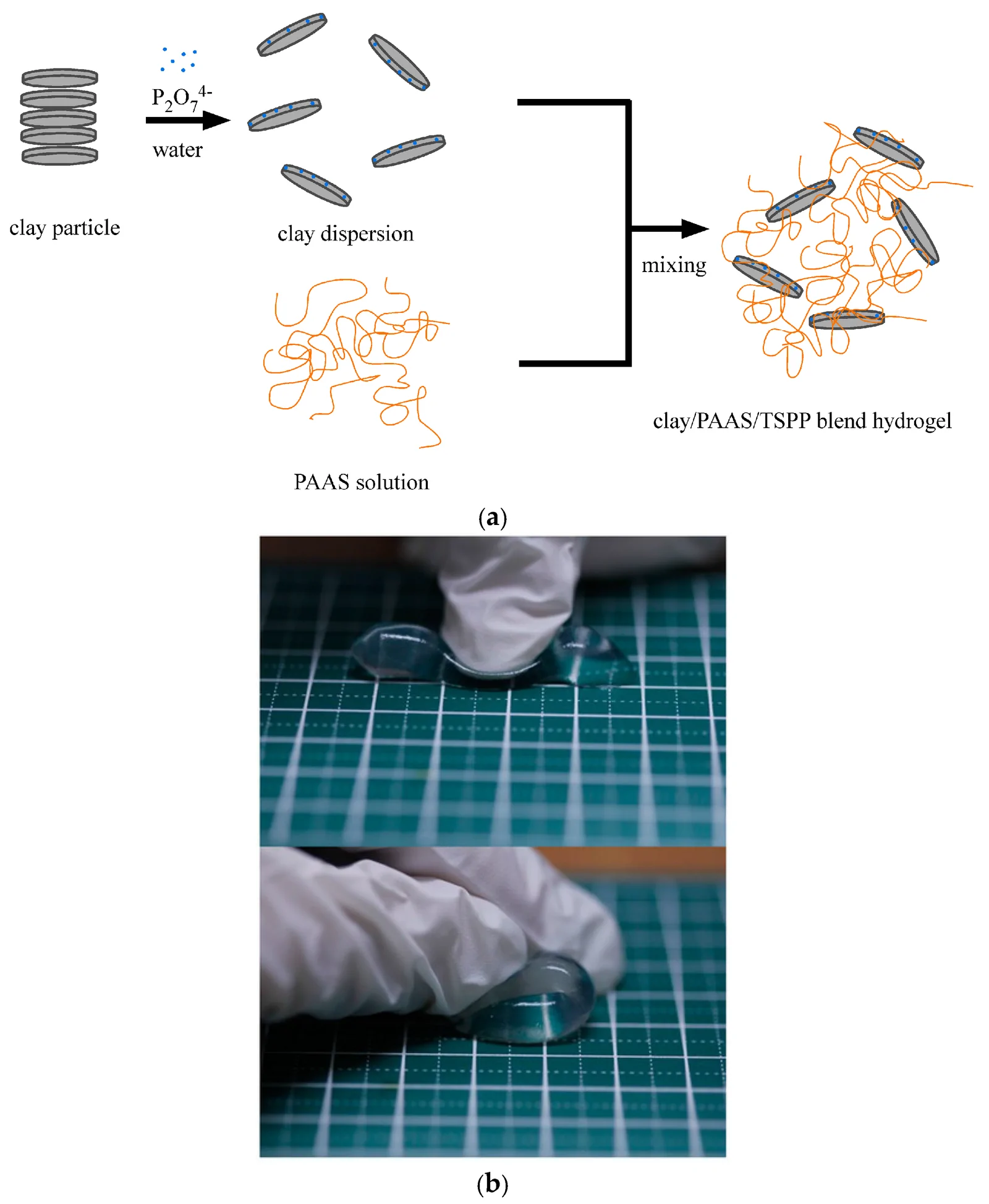
(**a**) A schematic representation for the preparation of clay/PAAS blend HGs, and (**b**) a polymeric hydrogel—a pressed gel (**top**) and a bent gel (**bottom**). Here, PAAS = polyacrylic acid sodium salt (or sodium polyacrylate), and TSPP = tetrasodium pyrophosphate. Reused from [67] under an open access license, MDPI.

**Table 1:** A comparison among three types of NC HGs.

Category	Carbon-Based NC HGs [35,36,40,42,43,45,68]	Metal-Based NC HGs [47,48,49,50,57]	Polymer-Based NC HGs [35,57,58,59,69]
Reinforcing materials	Graphene, CNTs, biomass-derived carbons	Ag, Cu, Zn NPs, metal oxides	Polymeric NPs, carbon-based NPs within polymer matrices
Structural features	3D hierarchical porous structure, high water retention	NP dispersion, tunable via metal concentration & NP size	Highly hydrated, porous 3D network mimicking biological tissues
Key properties	High electrical conductivity, mechanical robustness, tunability	Antibacterial, catalytic activity, electrical conductivity, mechanical strength	Flexibility, biocompatibility, biomimicry, multifunctionality
Biomedical applications	Wearable electronics, biosensing, human–machine interfaces	Wound healing, drug delivery, biosensing, tissue engineering	Regenerative medicine, cancer therapy, drug delivery, bio-actuation

## 3. Fabrication Techniques

### 3.1. In Situ Polymerization

In situ polymerization is a pivotal fabrication technique for NC HG sensors, enabling the direct formation of functional HGs embedded with NMs [70]. This method involves the polymerization of monomers directly within a host matrix, forming strong, uniform networks with improved NM dispersion (see Figure 3). It allows precise control over polymer growth and facilitates the integration of advanced functionalities, including self-healing, responsiveness, and enhanced mechanical properties, making it particularly valuable for applications in biomedicine, electronics, and nanotechnology [71,72,73,74]. During synthesis, nanofillers such as CNTs, cellulose nanocrystals (CNCs), metal–organic frameworks, or conductive polymers are incorporated into the HG matrix. Catalysts, such as tannic acid–silver complexes, or liquid metal/CNT systems are employed to accelerate polymerization, enabling rapid and energy-efficient fabrication [75,76,77,78].

For instance, Yu et al. [79] demonstrated the polymerization of calcium phosphate oligomers onto a PVA network using sodium alginate as a bonding agent. The resulting HG formed ultrafine hydroxyapatite nano lines (~1 nm diameter), exhibiting high mechanical strength (~17.84 MPa), fracture energy (~8.97 kJ/m^2^), and biomimetic motion, suitable for self-repairing biomedical applications.

Similarly, Zou et al. [80] developed organo-HG sensors via an autocatalytic system involving tannic acid (TA) and silver nanoparticles (AgNPs) on MXene nanosheets (see Figure 3), achieving rapid synthesis (10 min), exceptional stretchability (1740%), tensile strength (TS) (184 kPa), a gauge factor (GF) of 3.86, and high sensitivity (0.1% detection limit), ideal for monitoring human movement and physiological activity. The formation of a double-network system, achieved by combining two polymer networks (e.g., chitosan/polyacrylic acid) or integrating dynamic crosslinkers (e.g., Ag@polydopamine), further enhances the mechanical strength, stretchability, and self-healing capabilities of NC HGs [29,81]. Sun et al. [82] developed a double-network HG of polypyrrole and polydopamine (PDA) exhibiting a self-healing efficiency of 87.2%, tensile stress of 3.50 MPa, strain of 453.45%, and excellent sensing performance, including a gage factor (GF) of 9.9 and a fast response time of 260 ms—making it highly suitable for wearable electronics designed to monitor human motion. The primary advantage of in situ polymerization lies in its ability to achieve uniform NM dispersion and strong interfacial bonding, significantly enhancing both electrical and mechanical properties. However, it faces challenges, including the complexity in chemical reactions, control over reaction kinetics, and potential biocompatibility concerns due to residual reagents and O_2_ inhibition during processing [32,71].

**Figure 3:**
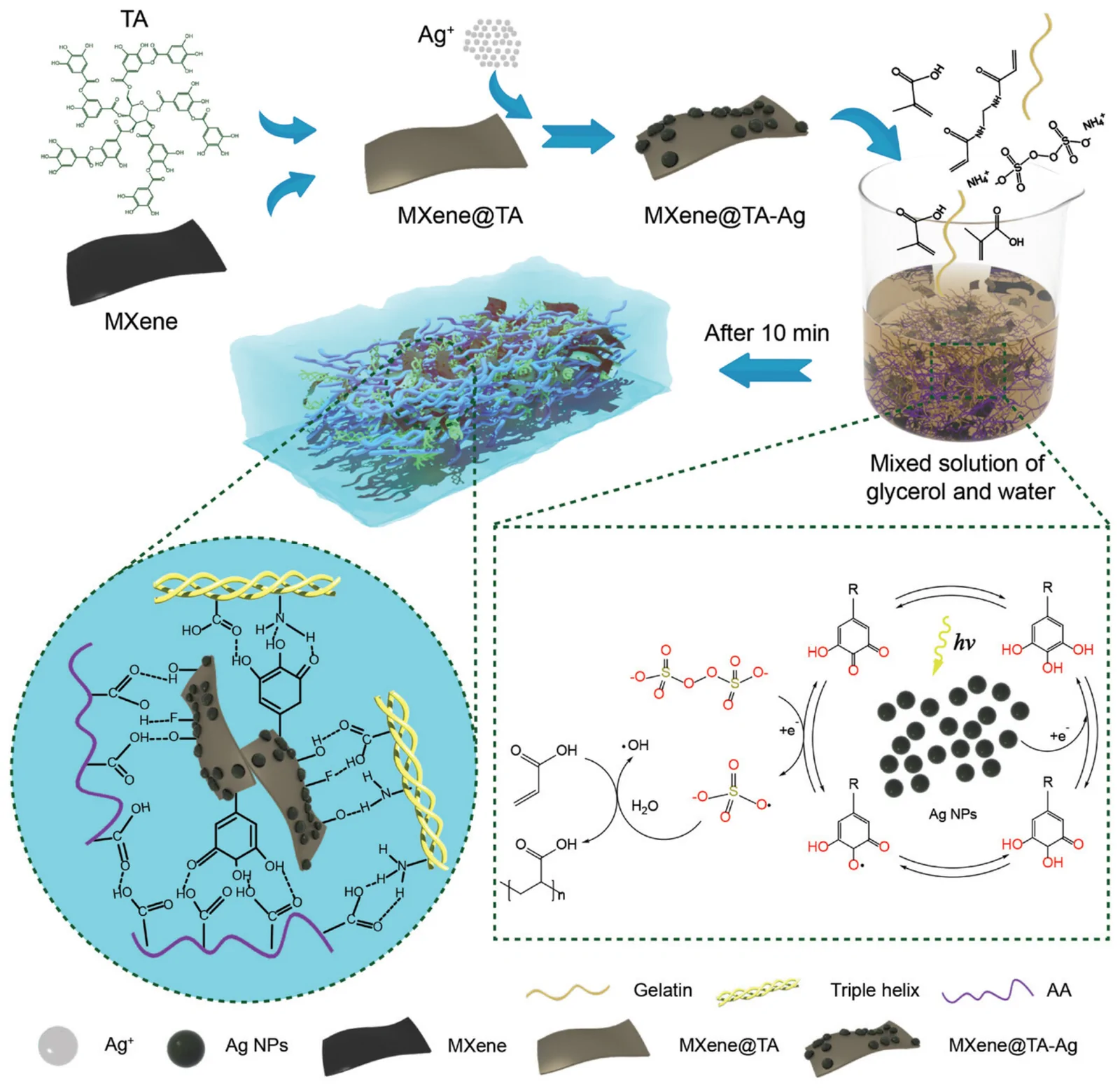
The formation process of the PMTAG organo-HG. Reused from [80] with permission from John Wiley and Sons, Inc.

### 3.2. Conformal Coating Process

Conformal coating involves applying a thin, uniform protective film over components, conforming to complex shapes and delicate structures. This technique provides electrical and mechanical isolation, protecting against moisture, dust, corrosion, and temperature extremes—widely used in automotive, medical and wearable electronics [83]. It fabricates conductive composites by first constructing a percolating network of NMs like CNF, MXene, or rGO and then infiltrating it with a HG matrix. This core-shell architecture ensures a continuous interface, yielding exceptional mechanical robustness and stretchability alongside high conductivity [11,84,85,86,87].

Shao et al. [84] utilized conformal coating of cellulose nanofiber (CNF) networks with supramolecular interactions involving polydopamine (PDA), Fe^3+^, and polyacrylamide to create self-adhesive epidermal sensors with excellent mechanical strength (tensile strength ~130 kPa, compressive strength ~10 MPa), degradability (30 days), and real-time motion-monitoring capabilities. Li et al. [85] fabricated conductive NC HGs by coating MXene (Ti_3_C_2_T_x_) into a polyacrylic acid–amorphous calcium carbonate matrix, resulting in stretchable, self-healing, fast-responsive (20 ms) and biodegradable HGs for epidermal sensors. Ma et al. [86] further enhanced HG performance by incorporating conductive NMs and anti-freezing agents into the H-polymer network. The NC organo-HGs are prepared from the conformal coating of functionalized GO network by the HG polymer networks consisting of PVA, phenyl-boronic acid grafted alginate, and polyacrylamide in the binary ethylene glycol (EG)/H_2_O solvent system. The obtained organo-HG exhibits excellent temperature tolerance (−40 °C), long-lasting moisture (20 days), improved electrical conductivity and mechanical strength for cold-weather wearable applications.

A major advantage of conformal coating techniques in NC HG-based wearable sensors is their ability to closely conform to the microtopography of human skin. This enhances user comfort, improves signal fidelity, and significantly reduces motion artifacts during dynamic activities [88]. Many conformal coating systems are designed to be biodegradable and non-toxic, thereby minimizing environmental and health risks following disposal [84,85]. These sensors are capable of monitoring a range of physiological parameters, including strain, pressure, and electrophysiological signals such as ECG and EMG. Additionally, some conformal coatings offer antibacterial and hemostatic functionalities, further expanding their utility in biomedical applications [87,88]. Despite these advantages, this technique faces several challenges. Ensuring long-term functionality, maintaining biocompatibility, and achieving stable performance under varying environmental and physiological conditions remain critical hurdles to their widespread adoption [89].

### 3.3. Dynamic Crosslinking of Polymers

Dynamic crosslinking of polymers is a versatile fabrication strategy (see Figure 4) that enables the formation of polymer networks exhibiting both thermoset and thermoplastic characteristics. This is achieved through reversible bonds that can break and reform under specific conditions, allowing for reshaping, recycling, and self-healing functionalities after the initial crosslinking stage [90,91]. These materials, known as covalent adaptable networks (CANs), offer tunable mechanical and structural properties, with the dynamic behavior of crosslinks often being temperature dependent. This responsiveness facilitates modifications in material characteristics and restoration of internal microstructure under defined environmental conditions [92].

Natural polymers like agarose, alginate, gelatin, chitosan, and casein can be dynamically crosslinked using various physical, chemical, or enzymatic methods to tune their mechanical properties and degradation rates [94,95]. Table 2 below correlates each polymer with its primary dynamic crosslinking methods, highlighting the underlying mechanism and key trade-offs.

In the context of NC HGs, dynamic crosslinking imparts essential properties such as self-healing, stretchability, and multifunctionality, which are critical for advanced healthcare sensor applications [101]. Dynamic covalent bonds, including borate esters, Schiff bases, and disulfide linkages, enable reversible bond formation and dissociation, thereby supporting self-repair and responsiveness to external stimuli [102,103,104,105]. In parallel, non-covalent interactions—such as H bonding, metal–ligand co-ordination (e.g., Fe^3+^), and host–guest complexes (e.g., cyclodextrin-ferrocene)—contribute to dynamic crosslinking that enhances HG elasticity, conductivity, and adhesion [106,107,108,109]. This approach has been employed to improve both mechanical and electrical performance by integrating components such as N, N′-bis(acryloyl) cystamine and silver-modified PDA NPs [29]. Redox systems, like lignin sulfonate combined with Fe^3+^ ions, have demonstrated exceptional stretchability and rapid self-healing capabilities [110]. Additionally, temperature-responsive HGs incorporating nanoclay and carbon black with N-isopropyl acrylamide adapt to thermal changes without mechanical deformation. These HGs exhibit impressive performance, including up to 98.8% damage repair within 15 s, strong adhesion to skin, high sensitivity (GF up to 11.63), and fast response times (as low as 138 ms), making them ideal for wearable applications [111,112]. However, challenges remain in optimizing biocompatibility and long-term stability for real-world healthcare use.

### 3.4. Combination of Foaming and Freezing Thawing

The combination of foaming and freezing–thawing cycles represents an innovative and effective fabrication strategy for NC HG sensors. This method is commonly employed to physically crosslink polymers such as PVA, where foaming introduces gas bubbles into the HG matrix, resulting in lightweight and porous structures. Concurrently, repeated freeze–thaw cycles induce phase separation, enhancing HGs’ elasticity, mechanical strength, and responsiveness—all achieved without the use of toxic chemical crosslinkers [113,114,115,116]. Successive freeze–thaw cycles further refine the pore architecture and improve network integrity, contributing to enhanced mechanical performance and long-term functional stability [117,118]. When combined, these processes yield NC HGs with excellent anti-freezing, self-healing, and water retention properties, enabling reliable operation across diverse environmental conditions [119,120]. For instance, Wang et al. [115] applied this dual-process technique to fabricate NC HGs with enhanced stretchability and compressibility. By fabricating a stretchable and dissolvable porous silver nanowires (Ag NWs)/PVA NC HG through a facile foaming and freezing–thawing combined approach, they produced flexible electrodes with high sensitivity (1.9 kPa^−1^), wide detection range (250 kPa), quick response time (25 ms), and remarkable stability over 10,000 loading/unloading cycles. The resulting HGs were environmentally friendly and capable of detecting human movements, demonstrating scalability for mass production.

The primary advantage of combining foaming with freeze–thawing lies in the creation of highly porous, mechanically robust, and responsive NC HG sensors, ideal for wearable health monitoring. However, this also presents challenges, including maintaining uniform pore distribution, preventing phase separation of components, and preserving electrical conductivity throughout the manufacturing process [121,122].

### 3.5. Self-Assembly

Self-assembly leverages H bonding, π−π stacking, and dynamic covalent or supramolecular interactions between NMs (e.g., cellulose nanofibers, CNTs, MXene, graphene, lignin NPs) and polymer matrices. These interactions create robust, conductive, and flexible networks [123,124,125,126]. Self-assembled NC HGs offer excellent flexibility, biocompatibility, and mechanical strength, making them suitable for wearable healthcare sensors (see Figure 3. These materials achieve high sensitivity (GF of 3.62 and 8.98), rapid response time (139 ms) and strong self-healing (up to 91.76% recovery within 2 h). Their adhesion to skin ensures comfort during prolonged wear [30,127]. Additionally, thermally responsive structural colors enable non-invasive temperature sensing, while reversible molecular interactions enhance stretchability. Despite these advantages, improving environmental resistance and long-term durability remains a key focus for future development.

### 3.6. Inkjet Printing

Inkjet printing is a cutting-edge, additive manufacturing technique that enables the precise, scalable, and customizable fabrication of NC HG sensors. It is a cost-effective and customizable method for fabricating wearable sensors, offering high-resolution patterning suitable for body-conforming designs. Inkjet printing can deposit nanostructured conductive HGs and multiple enzymes onto microelectrode arrays, enabling rapid, high-throughput production of multiplexed biosensors for simultaneous detection of analytes like glucose, lactate, and triglycerides [126,128]. NC inks are formulated with conductive NMs (e.g., MXene, PEDOT:PSS) and reinforcing agents (e.g., CNCs) to achieve the optimal balance of conductivity, mechanical robustness, and sensitivity required for printed electronics [129,130,131,132]. Inkjet-printed HGs can be integrated onto paper, polyamide, or microstructured HG surfaces, supporting flexible, wearable, and even low-temperature-resistant sensors [129,130,133]. It enables low-cost production and miniaturization of sensors for detecting subtle movements [134,135]. NC HGs used in this process are flexible and biocompatible, while conductive inks further enhance sensor performance [4,136,137]. These printed sensors can continuously monitor health signals and integrate data transmission features. However, ensuring long-term stability and environmental resilience is essential for reliable everyday use [4,134].

### 3.7. One-Pot Synthesis

One-pot synthesis is a streamlined fabrication method where all components—polymers, NMs, and functional additives—are combined in a single step, enabling efficient, scalable, and reproducible production of NC HG sensors. It simplifies the fabrication of NC HGs by combining multiple components in a single step, improving efficiency and material performance. This method often uses free radical polymerization to integrate polymers (e.g., polyacrylamide, PVA) with NMs like ZIF-8, cellulose nanofibers, or CNTs, resulting in HGs with enhanced mechanical strength, stretchability, and conductivity. Some systems generate NPs (e.g., gold, carbon dots) within the HG matrix during synthesis, imparting fluorescence, biosensing, or antibacterial properties [76,123,138,139,140,141]. For instance, a pseudo-slide-ring HG is synthesized by combining polyacrylamide (pAAm), β-cyclodextrin (β-CD), and poly 2(acryloyloxy)ethyltrimethylammonium chloride (AETAc) bio-ionic liquid (Bio-IL), achieving excellent stretchability (~3000%), strong mechanical toughness (1.1 × 106 Jm^−3^), tissue adhesion (~27 kPa), and good electrical conductivity (~0.29 S/m) without toxic reagents [142].

Techniques like cyclic freeze–thaw enhance sensitivity (4.013% kPa^−1^) and thermal stability [143], while nanozyme-catalyzed polymerization produces highly conductive, adhesive, and self-healing HGs suitable for harsh environments [127,144]. However, one-pot synthesis of NC HG sensors faces significant challenges in healthcare, including difficulty achieving uniform NM dispersion, robust mechanical properties, and consistent reproducibility. A single-step process must also overcome hurdles in ensuring biocompatibility, long-term stability in biological environments, and reliable sensor performance for safe and effective clinical use [145,146,147]. Despite these advancements and challenges, further work is needed to improve reusability and long-term performance for broader healthcare applications.

## 4. Multifunctionality of the NC HG Sensors

### 4.1. Mechanical Properties

NC HGs offer a compelling solution to the mechanical mismatch between rigid electronics and soft human skin by enabling conformal contact, thereby reducing interfacial impedance and skin irritation, which are critical considerations for wearable strain sensors. Conventional polymers are often brittle, while conductive filler-loaded elastomers may exhibit limited stretchability [148]. In contrast, NC HG-based sensors can combine flexibility, stretchability, toughness, and mechanical resilience, facilitating their integration with the human body for long-term healthcare monitoring. The incorporation of nanoparticles (NPs), including carbon-based materials, metal oxides, and polymer nanofillers, can reinforce HG networks, while biopolymers such as cellulose, chitosan, and silk fibroin can further improve biocompatibility and mechanical resilience [149]. Consequently, appropriately designed NC HGs can withstand repeated deformation without substantial deterioration of their sensing performance [150,151]. Representative tensile strength and stretchability values reported for different composite HG systems are summarized in Table 3.

The studies summarized in Table 3 demonstrate that mechanical performance can be tailored considerably through polymer-network design, intermolecular interactions, and functional reinforcement. For example, Xu et al. [152] developed a PVA/sodium glycinate (SG) HG in which strong H-bonding interactions and increased physical crosslinking generated by repeated freeze–thaw cycles enhanced mechanical integrity. Similarly, Hui et al. [153] employed H-bond engineering and multiple-network strategy in PVA/PEI/SG HGs, resulting in improved stretchability, toughness, fatigue resistance, and shape recovery. These findings indicate that strengthening reversible intermolecular interactions is an effective approach for improving mechanical durability without compromising the deformability required for wearable applications.

Alternative network-engineering approaches have produced even greater deformability. Liu et al. [154] used a “doping-then-gelation” strategy to construct a densely entangled polyaniline (PANI)/acrylic acid (AA) network that restricted PANI aggregation while providing high stretchability, self-healing capability, and strain sensitivity. Sun et al. [155] combined H-bonding and cation–π interactions in PAM-alginate/polypyrrole (PPy) HGs, achieving a favourable balance among mechanical robustness, sensing capability, and swelling resistance. Such supramolecular interactions are particularly attractive because reversible physical bonds can dissipate mechanical energy while maintaining network integrity during deformation.

Xu et al. [156] further demonstrated the versatility of network and molecular engineering by incorporating tannic acid (TA) and ionic liquids (ILs) into a p(AAm-co-PEGDA) HG. The resulting material exhibited remarkable deformability together with strong adhesion, illustrating how multiple functional components can simultaneously regulate mechanical and interfacial properties. The preparation process and major molecular interactions involved in this system are illustrated in Figure 5.

The systems listed in Table 3 also illustrate how nanofillers can introduce functionalities beyond conventional mechanical reinforcement. Liu et al. [157], for example, incorporated pyruvate-doped SiO_2_ and PPy into a dual-network PAM HG, producing a highly deformable material with adhesion, flame retardancy, and functionality under both cryogenic and ambient conditions. Wang et al. [158] incorporated AgNP-modified PDA@NFC into a PAM network, combining high stretchability with self-healing, antimicrobial activity, multimodal sensing, and cyclic durability. Thus, the mechanical data in Table 3 should not be considered independently; rather, they demonstrate how network architecture and nanofiller selection can provide a balance between strength, deformability, recovery, and additional functionality. This balance is particularly important for wearable sensors, where large and repeated deformations occur during practical use.

**Table 3:** Mechanical properties of composite HGs.

Composite	Matrix	TS (MPa)	Stretchability	Refs.
PVA/SG	PVA	1.16	300%	[152]
PVA/PEI/SG	PVA	2.2	386%	[153]
PANI/AA	AA	0.12	2830%	[154]
PAM-alginate/PPy	PAM	1.63	453%	[155]
SiO_2_/PPy (pyruvate-doped)	PAM	0.0797	1896%	[157]
PDA@NFC-AgNPs	PAM	>0.70	1100%	[158]

### 4.2. Electrical Conductivity and Sensitivity

Electrical conductivity and sensitivity are central functional characteristics of NC HGs used in actuators, biosensors, wearable electronics, and human-motion monitoring. Conductive HG-based sensors convert mechanical stimuli, including strain, pressure, and motion, into measurable electrical signals [159]. Conductive components such as metal NPs, MXenes (Ti_3_C_2_T_X_), carbon-based NMs (e.g., graphene, CNTs), metal ions, liquid metals, and conductive polymers can establish or reinforce charge-transport pathways within HG networks [159,160,161,162]. Representative conductivity and gauge factor (GF) values for these different conductive strategies are summarized in Table 4.

As shown in Table 4, no single conductive strategy provides an optimum combination of conductivity and sensitivity. Instead, the electrical response depends strongly on the type, distribution, and interaction of the conductive phase with the HG network. Carbon- and metal-based NMs generally form efficient percolating pathways, whereas metal ions, liquid metals, and conductive polymers provide alternative charge-transport mechanisms that can remain responsive during deformation. Notably, the reported systems show considerable variation in conductivity and GF, indicating that higher conductivity does not necessarily correspond to greater strain sensitivity. Therefore, the architecture and deformation response of the conductive network are as important as its intrinsic conductivity.

Hybrid metal/carbon nanostructures provide an effective route to multifunctional sensing. For example, AgNP-modified CNT networks combined strain sensing with antibacterial and temperature-responsive behavior [162], while other Ag-containing systems integrated conductivity with self-healing, antibacterial activity, and mechanical compliance [29,165]. Silver nanowire (AgNW)-based HGs further demonstrated that conductive networks can provide additional functionalities such as electromagnetic interference (EMI) shielding and self-healing [176]. These studies highlight the advantage of hybrid conductive networks when sensing performance and secondary functionalities are required simultaneously.

Ionic conduction provides a different mechanism through reversible ion transport and dynamic interactions within the HG network. Metal-ion-containing systems based on Al^3+^, Zn^2+^, Fe^3+^, and Cu^2+^ show a broad range of electrical responses (Table 4) [163,164,173,174,175]. In particular, PPy/CMC-Al^3+^/PVA HGs utilize the synergy between fixed conductive PPy domains and mobile ionic bridges [163], whereas cellulose-based Al^3+^/Zn^2+^ systems combine ionic conductivity with frost resistance and adhesion [173]. Such dynamic ionic interactions are advantageous for maintaining electrical functionality during large deformation and under variable environmental conditions. Carbon NMs and MXenes are particularly attractive because their high surface area facilitates the formation of interconnected electron-transport pathways. CNT-, reduced graphene oxide (rGO)-, and MXene-containing HGs generally exhibit favourable combinations of conductivity and GF (Table 4) [167,168,169,170]. For example, the synergistic interactions among rGO, PDA, and glycerol enabled sensitive and reversible strain detection [168]. However, the substantial differences among these systems demonstrate that filler type alone does not determine sensing performance; dispersion, interfacial interactions, network morphology, and deformation-induced changes in conductive pathways are also critical.

Conductive polymers such as PPy and poly(3,4-ethylenedioxythiophene) (PEDOT) provide another versatile approach because functionality can be integrated directly into flexible polymer networks [29,171,172]. PPy/PEDOT hybridization has produced highly conductive porous HGs suitable for biosensing [177], while self-doped PEDOT-S incorporated into a PVA/PAAm double network improved compatibility and electromechanical response [171]. These results demonstrate the potential of molecular and network-level engineering to overcome limitations associated with conventional conductive polymers. Beyond conductivity and GF, practical NC HG sensors increasingly require environmental tolerance and long-term stability. Water/glycerol-based HGs, for example, maintained stable electrical responses over a wide temperature range [178], while CNT-based piezoresistive HGs showed broad pressure detection and stable performance over repeated loading cycles [179]. Together with the self-healing and EMI-shielding systems discussed above, these findings indicate a shift from maximizing a single electrical parameter toward developing multifunctional sensors capable of operating reliably under complex service conditions.

Despite these advances, a fundamental trade-off remains between electrical and mechanical performance. Excessive conductive filler loading may improve charge transport but can cause aggregation, restrict polymer-chain mobility, and disrupt the HG network, whereas highly soft and stretchable networks may provide less stable conductive pathways. Strategies based on homogeneous nanofiller dispersion, reversible ionic interactions, supramolecular bonding, and salting-out effects can help mitigate this conflict [180]. Future NC HG design should therefore focus on optimizing the overall balance among conductivity, sensitivity, stretchability, mechanical durability, environmental stability, and long-term sensing reliability, rather than maximizing conductivity or GF independently.

### 4.3. Biocompatibility and Safety Properties

#### 4.3.1. Self-Healing Properties

In flexible electronics and wearable sensors, materials are frequently exposed to mechanical stress, friction, and environmental factors that can lead to damage and performance degradation. To address this, self-healing capabilities have become essential for extending the lifespan and reliability of NC HGs [181]. Self-healing HGs can autonomously restore their structure and functionality after damage, maintaining sensing performance and structural integrity over prolonged use. Self-healing mechanisms in NC HGs are typically categorized into 2 types:

(i) Stimuli-responsive healing, which requires external inputs such as heat, light, or chemical agents to trigger repair [182].
(ii) Autonomous healing, which relies on reversible interactions within HG matrix, such as dynamic covalent or non-covalent binding [183].

Incorporating NPs or self-healing polymers into the HG matrix enhances these capabilities. The healing process often involves dynamic covalent bonds (e.g., Schiff-base, disulfide, Diels–Alder reactions) or non-covalent interactions (e.g., H bonding, metal coordination, π–π stacking, ionic interactions, and host–guest chemistry) [183,184,185,186]. Figure 6 illustrates the rapid self-healing characteristics of HGs based on PVA/ε-poly-L-lysine (EPL)/hyaluronic acid (HA) [93]. After forming the dynamic network structure, the damage gap in the composite HGs can heal within 1 min, demonstrating excellent self-healing ability.

#### 4.3.2. Adhesion Properties

Self-adhesion is a critical feature for electronic skins and flexible strain sensors, ensuring stable and reliable contact with soft, irregular surfaces like human skin. Traditional HGs often rely on external adhesives (e.g., tapes or bandages), which can be bulky, poorly ventilated, and unsuitable for long-term wear. These external adhesives may compromise the durability, stability, and precision of HG-based sensors over time [187,188]. To overcome these limitations, researchers have developed inherently self-adhesive HGs that adhere directly to various substrates without aid. Inspired by mussel adhesion, catechol-functionalized HGs, primarily using TA and dopamine (DA), have gained prominence due to their strong interfacial bonding capabilities. TA is naturally abundant, cost-effective polyphenol with multiple catechol groups, making it ideal for biomimetic HG design [189].

Yan et al. [190] developed conductive NC HG using TA/PANI@CNC, Al ions, and functional acrylic monomers. The resulting HG exhibited >900% stretchability, >700 kPa TS, and long-lasting adhesion via borate bonds and supramolecular interactions. The NC HG can directly attach to fingers and withstand a stretching strain of 400% without the fixation of adhesive tapes. It also demonstrated self-healing within 30 min and high strain sensitivity, suitable for detecting subtle movements like pulses and droplets. Li et al. [75] created a PDA-modified CNT HG via in situ polymerization with acrylamide. The HG exhibited 97.3% self-healing efficiency, strong adhesion, and safe application on human skin. The NC HG-based strain sensor showed a high sense GF of 3.93 at a strain of 400% and responds quickly with a fast response time of 76 ms when detecting human movement. Bai et al. [191] developed an HG using in situ polymerization and freeze–thawing of dopamine within PVA, followed by the addition of conductive phytate. The resulting HG demonstrated enhanced ductility, biocompatibility, and adhesion, with phytic acid strengthening intermolecular interactions and PDA providing robust skin attachment. Xu et al. [192] synthesized a double-network HG using dopamine-modified carboxymethyl cellulose (CMC-DA), hexadecyl methacrylate and AM. The Hg achieved a TS of 210 kPa, adhesion of 83.2 kPa, conductivity of 8.75 mS/cm, and 2400% elongation. Its complexation with Fe^2+^/Fe^3+^ and hydrophobic interactions enabled consistent response and high strain sensitivity, making it ideal for wearable biosensors. Figure 7 presents photographs illustrating the (a) stretchability, (b) shapeability, and (c) adhesive properties of PBLH composite HGs fabricated by Sun et al. [93]. The HGs can be molded into a wide range of shapes (Figure 7b), and their length can be extended up to 15 times the original without any visible cracking, even under twisting deformation (Figure 7a). Furthermore, as shown in Figure 7b, the HGs can be readily reshaped into various forms, demonstrating their excellent ductility and structural adaptability. The HGs also exhibit strong adhesion to substrates with diverse surface characteristics, including steel, glass, plastic, wood, and paper cups (Figure 7c). Overall, the outstanding mechanical flexibility and tunable properties of the HGs indicate their strong potential for biomedical applications, particularly in wound dressing.

#### 4.3.3. Freeze Resistance and Water Retention/Reusability

HGs typically contain high-water content to maintain ionic conductivity and preserve their 3D structure. However, under extreme temperatures, water within HGs either freezes, leading to brittleness and loss of mechanical and electrical properties, or evaporates, compromising flexibility and functionality [193,194]. Water in HGs exists in 3 forms [195]:

(i) free water, which freezes at standard temperatures.
(ii) intermediate water, which binds weakly to polymer chains and freezes at lower temperatures.
(iii) bound water, stabilized by H-bonding and resistant to freezing.

To enhance freeze resistance and water retention, researchers have employed strategies such as:

(i) replacing free water with organic solvents (e.g., ethylene glycol (EG), glycerol (Gly) [196,197].
(ii) Incorporating ionic salts (e.g., LiCl, CaCl_2_, ZnCl_2_) [198].
(iii) Using biomass-derived antifreeze agents (e.g., phytic acid (PA), proline and betaine) [199].

These additives lower the freezing point, reduce vapor pressure, and inhibit ice crystal formation, thereby improving HG performance in extreme environments.

Bao et al. [197] incorporated silk nanofibers and graphite carbon nitride nanosheets into a PVA network using water–EG solvent. The HG maintained flexibility and conductivity at −18 °C, exhibited 99% antibacterial efficiency, and demonstrated fatigue resistance over 1000 cycles with stable performance for 20 days. Inorganic salts can disrupt H bonding in cellulose-based HGs, affecting mechanical strength. To counteract this, Liu et al. [198] dispersed cellulose in zinc salts and added attapulgite clay (ATP) to regulate cross-linking density. ATP improved porosity, reduced ion diffusion resistance, and enhanced freeze resistance and ionic conductivity. In another study, Liu et al. [199] synthesized CNC-g-GB by grafting betaine hydrochloride onto CNCs in a deep eutectic solvent integrated into an AGG/FeCl3/PAA matrix; the HG exhibited self-healing (86%), freeze resistance (−20 °C), and dynamic H bonding for water retention. Cai et al. [175] developed HGs using LiCl and alginate in an acrylic–acrylamide copolymer network. The HG demonstrated tensile strain up to 4500%, a GF of 3.59, and a broad detection range (0–4200%) for muscle movements.

### 4.4. Antibacterial Activity

NC-based HGs possess remarkable antibacterial properties that significantly enhance their suitability for wearable and biomedical sensors. These HGs integrate polymeric networks with embedded NMs, resulting in multifunctional materials that combine flexibility, electrical conductivity, and antimicrobial activity. Such features are essential for ensuring the safe and reliable operation of sensors in direct contact with human skin or biological environments [200,201,202].

The incorporation of NPs improves interactions with bacterial cells, thereby enhancing antibacterial performance. In addition, the porous structure of HGs facilitates bacterial entrapment and promotes close contact between bacteria and the embedded NPs, further improving antimicrobial efficiency [203]. Among the various antimicrobial fillers, silver nanoparticles/nanowires (AgNPs/AgNWs) are the most widely used due to their broad-spectrum antibacterial activity against pathogens such as *Escherichia coli* (*E. coli*) and *Staphylococcus aureus* (*S. aureus*). These systems commonly achieve antibacterial inhibition rates exceeding 99–100%, along with distinct inhibition zones [200,202,204].

Cheng et al. [200] developed a PKB/F-AgNPs HG with a hierarchical network structure, exhibiting 83% self-healing efficiency within 60 min and 128% cell viability in Cell Counting Kit-8 assays, indicating excellent biocompatibility (Figure 8). The HG showed strong antibacterial activity against *E. coli* and *S. aureus*, which was further enhanced under blue-light irradiation by 1.3-fold and 2.2-fold, respectively. Moreover, its antibacterial performance was monitored through changes in electrical conductivity, providing a cost-effective alternative to conventional microbial culture assays. These findings highlight the potential of antibacterial HGs for wearable electronics and human-machine interface applications.

Ag-based HG systems can additionally provide sustained antibacterial effects through the gradual release of Ag ions from the polymer network [201,205]. Beyond Ag-based systems, several alternative antibacterial strategies have also been explored, including Fe–tannic acid coordination NPs [204], cationic chitosan or dendrimers [202], tannic-acid-based chemistries [204], and PDA NPs [206]. For example, a composite HG fabricated from PVA, borate, EPL, and HA demonstrated promising antibacterial performance through the simple incorporation of EPL into the network. The resulting HG exhibited excellent antibacterial activity, achieving bacteriostasis rates of up to 90% against both *S. aureus* and *E. coli* [93]. In another study, a multifunctional HG based on MXene loaded with in situ-generated AgNPs was incorporated into a mixed guar gum/acrylic acid matrix. The resulting strain-sensing HGs exhibited outstanding antibacterial activity, achieving complete (100%) inhibition of both *E. coli* and *S. aureus* [207]. Table 5 documents the antibacterial effects and sensing functions of NC HGs.

### 4.5. Biodegradability and Environmental Impact

Beyond skin compatibility, the biocompatibility and safety of HG-based wearable devices should also be evaluated in terms of their interactions with cells and biological tissues, as well as the possible effects of their individual components, including polymers, crosslinkers, conductive fillers, and ionic additives. Natural polymers such as alginate, chitosan, and gelatin are generally attractive because of their renewable origin, biocompatibility, and biodegradability. For example, alginate is mainly derived from brown seaweed and can form biodegradable HG networks, making it a promising material for sustainable electronic and biomedical applications [209]. In addition, the use of natural and biodegradable polymers may reduce the environmental burden associated with the disposal of short-lifetime wearable devices and support the development of more sustainable and circular material systems [210].

Compared with renewable biopolymers, synthetic polymers can provide better control over mechanical properties, durability, and large-scale processing, but their environmental impact requires careful consideration [211]. PVA, for example, is widely used in wearable and biomedical HGs because of its hydrophilicity, low cytotoxicity, and favorable biocompatibility. However, its degradation strongly depends on its chemical structure and the surrounding environment, and its end-of-life behavior may be considerably slower than that of many naturally derived polymers [212,213]. In contrast, biopolymers such as alginate are obtained from renewable biomass and can undergo biological degradation, offering potential advantages in terms of raw-material sustainability and waste reduction [214]. Nevertheless, natural polymers may show lower mechanical stability and less controlled performance than synthetic polymers [215]. Therefore, the selection of HG materials for wearable electronics should consider not only biocompatibility and device performance, but also raw-material origin, biodegradability, waste generation, and the environmental effects of all device components throughout their life cycle.

## 5. Applications of NC HG Sensors in Healthcare

NC HG sensors are emerging as multifunctional platforms for healthcare because their soft, hydrated networks can conform to biological tissues, while NMs provide electrical, optical, mechanical, and biochemical functionality. This combination enables NC HGs to interface with physiological signals and convert them into measurable outputs. Their healthcare applications extend from routine monitoring of vital signs to chronic disease management, rehabilitation, continuous glucose monitoring, and intelligent wound care, as illustrated in Figure 9. The following sections discuss these applications, with emphasis on their practical functions and healthcare relevance, while quantitative performance characteristics are summarized in Table 6, Table 7, Table 8, Table 9 and Table 10.

### 5.1. Vital Sign Monitoring

NC HG sensors provide a soft interface between the human body and electronic monitoring systems, making them particularly attractive for continuous and wearable measurement of physiological signals. Their tissue-compatible modulus, stretchability, conformability, and biocompatibility facilitate intimate skin contact and enable detection of heartbeats, pulse, respiration, temperature, swallowing, facial movements, and other subtle physiological activities [216,217,218]. Representative sensing mechanisms and performance characteristics are summarized in Table 6.

Recent advancements demonstrate a transition from single-signal detection toward multimodal physiological monitoring. Piezoresistive NC HGs can detect small deformations associated with heartbeats, pulse, respiration, and muscle-related movements, while thermosensitive components enable simultaneous temperature monitoring [219,220]. Such multimodal capability could reduce the need for multiple independent wearable sensors and facilitate continuous personalized health monitoring.

NC HGs also offer opportunities for more specialized respiratory and neuromuscular monitoring. Moisture-responsive systems can detect changes in exhaled humidity and breathing patterns, providing a potential route for identifying abnormal respiration associated with sleep apnea [221]. Other highly deformable conductive HGs can capture swallowing, facial expressions, and related movements, with potential applications in muscle rehabilitation and human–machine communication [222]. Importantly, antibacterial and antioxidant components such as chitosan and polydopamine can improve suitability for prolonged epidermal contact [220,222]. Thus, the key advantage of NC HGs for vital-sign monitoring lies not only in sensitivity, but in their ability to combine conformal sensing, multimodal detection, environmental tolerance, and skin-compatible functionality within a single wearable platform.

**Table 6:** Applications of NC HG sensors in vital signs monitoring.

Vital Sign	NC HGs	Sensing Mechanism	Physiological Performance	Application Status	Refs.
Heartbeat & Temperature	SA-PBAD@PHEAA (Na alginate/boronic acid/acrylamide)	Dual-mode: piezoresistive geometric modulation of ionic channels and thermoresistive activation of ion mobility	GF of 5.17; TCR of −3.48%/°C (0–20 °C); 6570% elongation; 104 ms response time	Personalized health monitoring & electronic skin in extreme environments (−70 to 80 °C)	[219]
Pulse & Respiration	Ag@PDA/(CSMA-PAM) (Ag nanodots/polydopamine/chitosan/acrylamide)	Piezoresistive: directional drift of free electrons in Ag nanostructures combined with Schiff base linkages	GF of 2.13; 0.5% strain detection limit; 550 ms response time; hemolysis ratio < 5%	Epidermal sensing of rat pulse (63–87 bpm) & human breathing patterns	[220]
Swallowing & Smiling	PGCN (PAM/Gelatin/c-MWCNTs/Chitosan)	Piezoresistive: synergistic electron/ion conduction via multiwalled CNTs & inorganic salt electrolytes	Conductivity of 5.09 S/m; GF of 1.91; retains 60% toughness after 10 cycles at 1000% stretch	Remote communication (Morse code) & clinical muscle rehabilitation	[222]
Sleep Apnea (SAHS)	PVA-GO/LiBr (PVA/GO/lithium bromide)	Self-powered: moisture-triggered ion mobility via metal–air redox reaction	Sensitivity of 0.299 nA/s per %RH range; high ionic conductivity of 0.33 S/cm	Integrated telemedicine platform for real-time apnea diagnosis & medical management	[221]

### 5.2. Chronic Disease Management

Beyond routine physiological monitoring, NC HGs are increasingly being explored for continuous disease surveillance and combined sensing-therapy platforms. Their ability to respond to biological, electrical, optical, and environmental stimuli enables applications in diabetes management, cancer treatment, cardiac repair, respiratory monitoring, metabolic-health assessment, and diabetic wound care. Representative systems and their quantitative performance are summarized in Table 7.

Diabetes represents one of the most promising application areas. Glucose-responsive NC HGs can operate through electrochemical, optical, or fluorescence-based mechanisms and have been developed for continuous or minimally invasive monitoring in interstitial fluid, tears, and wounds [219,220,223].

Importantly, these platforms demonstrate how NC HGs can move beyond simple glucose detection. For example, wound-oriented systems can simultaneously monitor glucose and pH while supporting angiogenesis and tissue repair [223]. Such integration of diagnosis and therapy is particularly attractive for chronic diabetic wounds.

NC HGs are also being investigated as therapeutic platforms. Stimuli-responsive GO-Fe_3_O_4_/polymer HGs enable controlled anticancer drug release in response to near-infrared (NIR) irradiation, magnetic fields, and tumor-associated conditions [224]. In cardiovascular applications, conductive HGs can electrically bridge damaged myocardial tissue and support restoration of electrophysiological function [225]. These examples represent an important evolution from passive biosensors toward theranostic NC HGs, in which monitoring and therapeutic intervention are incorporated within the same material platform.

Respiratory and metabolic applications further broaden their clinical potential. Self-powered humidity/strain sensors can monitor abnormal breathing patterns associated with sleep apnea [221], while electrochemical NC HG platforms can detect metabolites such as uric acid in sweat [216]. Collectively, these studies indicate that the major advantage of NC HGs in chronic disease management is their capacity to combine continuous biomarker monitoring, minimally invasive sensing, responsive therapy, and tissue-compatible interfaces. However, most systems remain at laboratory, in vitro, or small-animal validation stages, and translation to long-term human use will require further assessment of biocompatibility, calibration stability, reproducibility, and device integration.

**Table 7:** Applications of NC HG sensors for chronic disease monitoring.

Chronic Disease	HG NCs	Sensing Mechanism	Physiological Performance	Application Status	Refs.
Diabetes Mellitus	PBA/Pro/SF-MN (phenylboronic acid on proline-treated silk fibroin microneedles)	Enzyme-free electrochemical sensing where PBA reversibly reacts with glucose, increasing electronegativity & altering current	Sensitive linear response (1–20 mM); sensitivity of ~0.5 μA/mmol (1–12 mM) and ~1 μA/mmol (12–20 mM)	Continuous monitoring for up to 21 days; demonstrated high stability against environmental pH & temperature	[219]
Cancer (Tumor Treatment)	GO-Fe_3_O_4_ /PNIPAM-AAm/alginate (GO-magnetite in interpenetrating polymer)	Multi-responsive release; NIR light & magnetic fields generate heat, causing PNIPAM to deswell & release doxorubicin (DOX)	Cumulative release of 86.1% DOX at pH 5.0 (tumor condition) vs. 44% at pH 7.4 over 78 h	In vitro cytotoxicity assays confirmed effectiveness in killing MCF-7 cancer cells	[224]
Myocardial Infarction (MI)	AT-EHBPE/HA-SH (conductive hydrogel anchoring stem cell exosomes)	Electrical integration; the conductive matrix bridges infarcted tissue to restore synchronous contraction & electrophysiological conduction	Electrical conductivity of 6.3 × 10^−4^ S/cm, closely matching native myocardium	In vivo evaluation in rat models over 28 days showing improved ejection fraction & reduced fibrosis	[225]
Sleep Apnea (SAHS)	PVA-GO/LiBr	Self-powered dual-mode; humidity-triggered metal-air redox reaction generates current, while resistance variations detect strain/breathing	Humidity sensitivity of 0.299 nA/s per %RH; resistance increases 6-fold at 400% tensile strain	Integrated into a respiratory monitoring platform for real-time detection of abnormal breathing patterns	[221]
Diabetic Wounds	PF127@Zn/C-G (carbon-dot-Zn^2+^ assembly in HG)	Visual monitoring; GOx-catalyzed oxidation of glucose generates acid that decomposes the matrix, activating carbon dot fluorescence	Glucose detection limit (LOD) of 0.15 mM; high predictive accuracy for wound pH via machine learning (96%)	In situ monitoring of biomarkers (pH & glucose) while promoting angiogenesis & wound closure in murine models	[223]
Metabolic Health (Uric Acid)	PVA/CNCs@PDA-Au	Electrochemical redox; specific reaction of uric acid at the HG working electrode generates a measurable current signal	Wide linear range (1.0–100.0 μmol/L) with a low detection limit of 0.42 μmol/L	Demonstrated dual detection of human joint motion & sweat uric acid; 10-day stability recorded	[216]
Diabetes (Tear Glucose)	Ag NPs/S-CNF/GO/4-MPBA/PVA (silver-cellulose-GO SERS platform)	SERS-based sensing; ratiometric Raman intensity shifts occur when 4-MPBA specifically binds tear glucose to form borate	Ultra-low detection limit of 1.627 × 10^−10^ M for glucose	Non-invasive mini-eye patch; validated using human tear fluid with recoveries between 90% & 110%	[220]

### 5.3. Rehabilitation and Physical Therapy

Rehabilitation requires continuous assessment of body movement, joint loading, muscle activity, and recovery progress. Conventional rigid sensors can restrict movement or lose conformal contact during repeated deformation, whereas NC HG sensors can accommodate stretching, bending, compression, and complex body motions. Their mechanical compliance and electromechanical responsiveness therefore make them particularly suitable for motion tracking, gait analysis, electrophysiological monitoring, sports rehabilitation, and assistive communication. Representative systems are compared in Table 8. Strain-responsive NC HGs can translate joint and muscle movements into electrical resistance changes, enabling real-time monitoring during rehabilitation exercises [147,226,227]. MXene-containing HGs further extend this capability to weak electrophysiological signals, supporting applications such as EMG/EOG monitoring, eye tracking, sign-language recognition, and body-temperature monitoring [226]. Self-healing and tissue-adhesive HGs are particularly advantageous for repeated rehabilitation activities because they can maintain conformal contact and recover functionality after mechanical damage [107,227].

Pressure-sensitive NC systems provide another important application route. Iontronic sensors can map plantar pressure during walking, enabling quantitative gait analysis, injury prevention, rehabilitation training, and sports-performance assessment [228]. Meanwhile, anti-swelling HGs have enabled underwater gait recognition, demonstrating potential for aquatic rehabilitation environments [229]. Integration with machine-learning algorithms further allows complex movement patterns to be classified rather than simply detected.

Overall, NC HGs could transform rehabilitation sensors from passive motion detectors into interactive rehabilitation interfaces capable of tracking movement, electrophysiological activity, gait patterns, and therapeutic progress in real time. Their future clinical value will depend on long-term cyclic stability, signal repeatability, user comfort, and reliable interpretation of complex physiological signals.

**Table 8:** Applications of NC HG sensors for rehabilitation and physical therapy.

Rehabilitation & Physical Therapy	HG NCs	Sensing Mechanism	Physiological Performance	Application Status	Refs.
Real-time motion tracking in sports & rehabilitation	PAM/PVA/BC4-NaCl20	Resistance-based strain sensing; mechanical strain alters the conductive network, leading to measurable resistance variations correlated with joint movements. Uses Na^+^ & Cl^−^ ions for conductivity via mobile ion transport	Fracture stress of 406 kPa & an elongation at break of 1019%	Health monitoring, physical rehabilitation & sports analytics application	[147]
Healthcare monitoring & physical rehabilitation	PAM/(MXene-SF)	High strain sensitivity, enabling precise monitoring of human motion & weak electrophysiological signals like electromyograms and electrooculograms	High stretchability (1560%), toughness (165 kJ/m^3^), & conductivity (0.25 S/m)	Sign language translation, eye tracking, & body temperature monitoring	[226]
Real-time human motion monitoring	PVA/MPS@PDA-Au	Strain sensitivity, where the HG detects changes in resistance when subjected to deformation, allowing for the monitoring of human movement & subtle physiological signals	High deformability (508.6%), mechanical strength (2.9 MPa), & self-healing properties (90.5%), along with fast response time (123.2 ms)	Real-time human motion monitoring, ascorbic acid detection in sweat, & electrophysiological signal monitoring	[227]
Health, injury prevention & rehabilitation	Polyimide-based integrated iontronic flexible pressure sensor	Iontronic capacitive sensing	High sensitivity (158.67 kPa^−1^), an ultrawide pressure sensing range (0–4000 kPa), low detection limit (0.25 Pa), fast response time (2.4 ms)	Plantar pressure monitoring, gait analysis, sports performance optimization & rehabilitation training	[228]
Human motion monitoring	PLOD (Poly(OEGMA-co-DEGMA) crosslinked with Laponite^®^ (LAP) and PEDOT:PSS)	Strain sensing	Excellent stretchability (~450%), high self-healing efficiency (~95%), tissue adhesion force (~0.5 N/cm^2^)	Triboelectric nanogenerators	[107]
Real-time monitoring of human movement	NCNW (noncovalent-water hydrogel)	Strain sensing	TS = 2.5 MPa, toughness (3.98 MJ/m^3^), low hysteresis (91.1% recovery after 30 s)	Underwater gait recognition systems	[229]

### 5.4. Real-Time Glucose Monitoring for Patients

Continuous glucose monitoring (CGM) is a particularly important application of NC HG sensors because HG networks can establish conformal contact with skin or biological fluids, while glucose-responsive NMs provide optical or electrochemical transduction. Current approaches include optical/plasmonic sensors, microneedle-based electrochemical sensors, nanofibrous platforms, and multifunctional wearable patches, as summarized in Table 9.

Optical NC HG sensors exploit glucose-induced changes in HG swelling or molecular interactions to alter plasmonic or optical signals. PBA-functionalized and Au NP-containing systems have demonstrated rapid and reversible glucose detection, offering potential for compact and remotely readable CGM devices [230,231]. Their principal advantage is the possibility of optical detection without conventional electrical sensing circuitry. Microneedle NC HGs provide a complementary minimally invasive strategy by accessing dermal interstitial fluid rather than relying on blood sampling. Conductive nanometal and PEDOT microneedles can perform electrochemical glucose detection while HG layers facilitate fluid extraction and tissue-compatible contact [232,233]. Integration with wireless electronics further creates opportunities for real-time transdermal monitoring. Nanofibrous HGs extend the concept toward less invasive skin-mounted systems. Enzyme-functionalized conductive nanofiber networks can extract and analyze interstitial glucose, while mechanically robust hybrid HGs can simultaneously detect body motion and glucose, suggesting that future NC HG-based CGM devices may combine glucose sensing, motion monitoring, wireless communication, and wearable integration within a single platform.

Despite promising sensitivity and response characteristics (Table 9), practical CGM requires more than a low detection limit. Long-term operations must address sensor drift, biofouling, selectivity against interfering biomolecules, repeated calibration, enzyme stability where applicable, and reliable correlation between interstitial or tear glucose and clinically relevant blood-glucose levels. Therefore, future development should emphasize continuous in vivo reliability and patient usability, rather than further improvement of analytical sensitivity alone.

**Table 9:** Applications of NC HG sensors in real-time glucose monitoring for diabetes patients.

Real-Time Glucose Monitoring	HG NCs	Sensing Mechanism	Physiological Performance	Application Status	Refs.
Continuous glucose monitoring	PBA-based HG functionalized with phenylboronic acid	surface plasmon resonance	high sensitivity (1.1% mM^−1^), a rapid response time (30 s), a detection limit of 1 mM	continuous glucose monitoring in diabetic patients,	[230]
Continuous glucose monitoring	Au NP incorporated HG optical fiber sensor	localized surface plasmon resonance	high sensitivity, rapid response time (30 s), reproducibility, & a detection limit of 1 mM	non-invasive glucose monitoring in diabetic care	[231]
Real-time glucose monitoring	Opamine-hyaluronic acid HG embedded with PEDOT:PSS & Ag-Pt nanoparticles	nonenzymatic electrochemical sensing	high conductivity, rapid response, reproducibility & a limit of detection (LOD) of 0.9 mM	real-time glucose monitoring in diabetic rats	[233]
Continuous glucose monitoring	PVA/BTCA/β-CD/GOx/AuNPs nanofibrous HG	amperometric glucose sensing	High enzyme activity (76.3%), excellent mechanical properties (12.1 MPa in dry state, 5.33 MPa in wet state), high water absorption & fast response times (<15 s)	invasive continuous glucose monitoring in human interstitial fluid	[234]
Human-body motion	Hierarchical hybrid NC HG	piezoresistive & amperometric sensing mechanisms	high mechanical strength (7.7 MPa), toughness (7.48 MJ/m^3^) & electrical conductivity (0.14 S/m)	human-body motion detection, non-invasive glucose monitoring	[10]

### 5.5. Wound Healing and Management

NC HGs are particularly well suited to wound care because the same hydrated material can serve as a protective dressing, sensor, and therapeutic delivery platform. Smart HG dressings can monitor wound-associated parameters such as pH, temperature, exudate, biochemical changes, and local mechanical signals while simultaneously maintaining moisture balance and delivering antibacterial or regenerative agents. Representative systems and their functional characteristics are summarized in Table 10.

One major application is the early identification of infection and abnormal healing. pH-responsive bacterial-nanocellulose HGs can provide visual or colorimetric information on changes in the wound environment, while preserving the conformability, water retention, and vapor transport required for wound dressings [235]. Other NC HG systems integrate pH, temperature, exudate, and mechanical monitoring with antibacterial, antioxidant, hemostatic, or gene-regulatory functions [236]. These multifunctional platforms could therefore provide information about wound status without repeatedly removing the dressing.

A second important direction is closed-loop or remotely connected wound management. Conductive multilayer HGs can convert changes in wound exudate into electrical signals and potentially transmit alerts indicating when a dressing requires replacement [237]. Integration with wireless communication could reduce unnecessary dressing changes and support remote management of chronic wounds. NC HGs can also combine sensing with active treatment. NM-containing dressings can provide antimicrobial activity, controlled drug release, gene regulation, and support for tissue regeneration [236,237,238]. Conductive HGs additionally offer the possibility of monitoring local motion and electrophysiological signals around the wound region [75,85,239]. Thus, NC HGs are evolving from passive wound coverings toward intelligent wound-care systems capable of sensing → reporting → responding to the wound microenvironment.

The major translational challenge is to demonstrate that these multifunctional systems remain accurate and safe in complex wound environments containing proteins, enzymes, microorganisms, changing pH, and variable exudate levels. Long-term biocompatibility, sterilization, adhesion without secondary tissue damage, sensor stability, and clinical validation therefore remain important priorities before widespread healthcare implementation.

**Table 10:** Applications of NC HG sensors in wound healing and management.

Wound Healing & Management	HG NC	Sensing Mechanism	Physiological Performance	Application Status	Refs.
**Real-time wound status monitoring**	TA-siRNA@BSA	Dual crosslinking network	high tissue adhesion, enhanced swelling (4.45-fold), superior mechanical strength, high siRNA release	real-time wound monitoring, infected wound management, gene silencing & vascular tissue regeneration	[236]
**Chronic wound management**	pH-responsive bacterial nanocellulose-based HG	Colorimetric sensing mechanism	high conformability, excellent mechanical strength, good water retention capacity & ideal water vapor transmission rates	H-responsive wound dressings, enabling early-stage infection detection, wound healing monitoring	[235]
**ECG signal monitoring**	MXene-PAA-ACC	Strain-sensing mechanisms	high mechanical strength (450% elongation), self-healing ability (healing time of 0.2 s)	real-time human motion detection, EMG & ECG signal monitoring	[85]
**Personalized healthcare**	PDA@CNT/PAM	Triboelectric effect & piezoresistive effect	self-heal with an efficiency of up to 97.3% after damage	motion detection, health monitoring & emotional expression transmission	[75]

The following Table 11 provides a comprehensive overview of nanocomposite hydrogel sensor performance across various healthcare applications.

## 6. Sustainability

HG sensors can reduce some e-waste by replacing rigid electronics with soft, wet materials, yet their environmental profile depends on polymer choice, fillers (e.g., various NMs), and degradation by-products, which may be toxic if not controlled [240]. Recent demonstrations show degradable NC HGs that can depolymerize under mild conditions (e.g., water, H_2_O_2_, enzymatic or Fenton-like chemistries), enabling transient epidermal or implantable sensors with reduced persistent waste [84,85,241]. Key environmental concerns include the persistence of synthetic polymers and conductive fillers (e.g., some carbon, metal or MXene residues) and potential toxicity of degradation by-products, which require explicit assessment during design [240,242]. Some studies have shown that cellulose nanofibers and catechol/Fe^3+^-based NCs degraded in ~30 days in water via Fenton-like reactions [84], while MXene-PAA/calcium carbonate HGs degraded in PBS or H_2_O_2_, reported as not polluting the environment in lab tests [85]. Hence, selecting biodegradable matrices and designing controlled degradation pathways (programmable kinetics, benign by-products) are essential to minimize ecosystem and human-health impact [84,240,241,242]. Also, natural polymers (cellulose, bacterial cellulose, chitosan, silk) and biodegradable or recyclable synthetic polymers and conductive components can be used for sustainability. In the case of manufacturing practices, more sustainable synthesis routes should be preferred, e.g., bio-mediated NP formation and solvent-minimizing processes [143,243,244,245].

The biocompatibility of HG sensors is tied to the same material and degradation factors. Epidermal HG sensors should be non-cytotoxic, breathable, mechanically compliant and non-irritating for long-term use [84]. However, implantable HG sensors require more rigorous evaluation. This is because, when implantable HG sensors contact tissue, they may cause inflammation, biofouling, fibrous encapsulation or foreign-body responses [85]. Therefore, appropriate polymers, low-toxicity crosslinkers and conductive fillers, tissue-matched mechanical characteristics and controlled degradation are needed. Moreover, long-term studies assessing cytotoxicity, local inflammatory responses, degradation products and functional stability in physiologically relevant settings are required [86].

Sustainable end-of-life (EoL) management of NC HG sensors is a growing research interest in the recent era [240]. In addition, environmental performance of the materials must be studied via life cycle assessment (LCA) methodology to understand the sensor products’ environmental friendliness over their life cycle [216,241]. The challenges of these materials against the regulatory and standardization issue are discussed in the following sections (Section 7.3).

## 7. Challenges

NC HG-based wearable sensors offer multifunctionality, flexibility, and biocompatibility, making them promising candidates for healthcare applications. However, several technical and practical challenges must be addressed to ensure their long-term reliability, scalability, and performance.

### 7.1. Technical Challenges

Despite the promising potential of NC HG-based sensors in wearable healthcare applications, several technical challenges must be addressed to ensure their long-term functionality, reliability, and scalability. A common limitation is the brittleness and poor mechanical resilience of many NC HGs, which restricts their durability under repeated mechanical strain. Additionally, water loss over time compromises flexibility and degrades sensor performance in wearable electronics [123]. Insufficient adhesion to skin and lack of self-healing capabilities further reduce sensor reliability during movement. Enhancing interfacial bonding and integrating autonomous repair mechanisms are therefore critical for long-term wearability [123].

Achieving multifunctionality, particularly balancing mechanical strength, flexibility, and electrical conductivity, remains a significant challenge. For example, chitosan/polyacrylic acid double-network HGs require optimization to simultaneously support strain sensing and self-healing [81]. Incorporation of NMs such as CNTs and carbon black into PVA/gelatin matrices has shown improvements in mechanical performance for wearable applications [246], while TEMPO-oxidized cellulose nanofibers crosslinked with CNTs have demonstrated improved flexibility and antifatigue behavior [123]. Environmental factors also pose challenges. Exposure to low temperatures can degrade HG properties, leading to reduced conductivity and mechanical failure [86]. Enhancing freeze tolerance and dehydration resistance is essential for real-world deployment. Many HGs also suffer from limited fatigue resistance, degrading within weeks of use. Triple-network HGs incorporating Ti_3_C_2_Tx nanosheets have shown promise in improving mechanical pliability and strain responsiveness [247].

For biomedical applications, NC HGs must be non-toxic and biocompatible. Inadequate biocompatibility can lead to adverse reactions, limiting their use in implantable or skin-contact devices [248]. Addressing the chemo-chemical mismatch between sensors and biological tissues is critical. Microgel-based crosslinking strategies have been proposed to improve modulus, fatigue strength, and elongation, supporting their use in implantable systems [248]. From the manufacturing perspective, the multi-step synthesis of NC HGs presents scalability challenges. Developing cost-effective and sustainable fabrication methods is necessary to support commercial production and widespread adoption. Additionally, phase segregation and conductivity loss in ionogels remain problematic. Recent advances incorporating ionic liquids and clay nanosheets have improved mechanical strength and conductivity under harsh conditions, making these materials more suitable for long-term use [249].

### 7.2. User-Related Challenges

For NC HG-based wearable sensors to be effectively adopted in healthcare, they must meet user-centric requirements, including comfort, safety, ease of use, and long-term wearability. Several challenges persist in optimizing these aspects. To ensure safe and prolonged skin contact, sensors must be soft, breathable, and non-irritating. Materials such as silk fibroin and bio-adhesive HGs have demonstrated potential in minimizing skin reactions and enhancing breathability [250,251]. Self-adhesive HGs that gently adhere to the skin without causing discomfort or leaving residue are essential for daily use, improving user compliance and reducing the risk of skin damage [250,252]. HGs must retain moisture, flexibility, and functional integrity over extended periods. Certain organo-HGs have shown the ability to maintain performance for over 20 days, making them suitable for continuous physiological monitoring [248]. Self-healing HGs further reduce maintenance requirements, while robust mechanical properties ensure durability during routine activities such as exercise and sleep. Wearable sensors should also be capable of monitoring multiple physiological signals (e.g., temperature, strain, glucose) while remaining responsive to environmental changes. Anti-freezing HGs, for instance, maintain performance under low-temperature conditions, thereby enhancing their practicality in diverse settings [85,167,251]. Achieving long-term wearability, multifunctionality, and low maintenance remains a critical challenge in the development of user-friendly NC HG-based wearable sensors for healthcare applications.

### 7.3. Regulatory and Standardization Issues

As NC HG-based wearable sensors continue to advance in healthcare, addressing regulatory and standardization challenges is essential for their safe and effective integration into clinical practice. Sensors must undergo rigorous testing for toxicity, skin compatibility, and long-term stability to meet medical device safety standards. Materials like silk fibroin and chitosan require validation to ensure they do not provoke adverse biological responses [250,252].

Environmental sustainability is increasingly emphasized by regulatory bodies. The development of biodegradable HGs is being explored to reduce electronic waste and align with global sustainability goals [84,85]. Sustainability issues are briefly discussed in the previous section (Section 6). Data privacy and security are also paramount. Wearable sensors must comply with data protection regulations such as GDPR and HIPAA. End-to-end encryption and secure data transmission protocols are critical to protect patient health information [10,86]. Material variability and synthesis complexity pose additional challenges. Differences in the properties and fabrication methods of NMs (e.g., MXene, GO, and silver nanowires) complicate standardization and scalability. Uniform performance metrics and reproducible fabrication processes are necessary to ensure consistency across sensor platforms [115,251]. Establishing standardized criteria for evaluating sensitivity, response time, mechanical properties, and biocompatibility is critical for comparative analysis and quality assurance [81,85]. Furthermore, sensors must support standard communication protocols compatible with electronic health record (EHR) systems to enable seamless data exchange and integration into existing healthcare infrastructures [85]. Addressing the regulatory and standardization issues is vital for the clinical translation and widespread adoption of NC HG-based wearable sensors in modern healthcare systems.

## 8. Conclusions

The integration of nanocomposite hydrogels (NC HGs) into wearable sensor technologies represents a significant advancement in healthcare monitoring. By leveraging the unique properties of carbon-based, metal-based, and polymer-based NCs, these HGs offer enhanced biocompatibility, flexibility, and stretchability—key attributes for continuous, real-time physiological monitoring. The high water content and self-healing capabilities contribute to long-term durability and reliability, making them well-suited for extended use in clinical and non-clinical settings. Fabrication techniques such as in situ polymerization, conformal coating, and dynamic crosslinking have demonstrated substantial improvements in sensor performance and scalability, facilitating their practical deployment across diverse healthcare applications. NC HG-based sensors have shown particular promise in chronic disease management, rehabilitation, and personalized health monitoring, where accurate and timely data acquisition is critical. Their strong adhesion to skin and resistance to environmental stressors further ensure consistent performance under real-world conditions.

Looking ahead, future research should focus on several key areas to fully realize the potential of NC HGs in next-generation healthcare technologies. These include:

- Enhancing mechanical and electrical properties to improve sensor sensitivity, durability, and responsiveness.
- Optimizing biocompatibility and biodegradability to ensure safety and sustainability in long-term and implantable applications.
- Developing scalable and cost-effective fabrication methods to support commercial production and widespread adoption.
- Establishing standardized performance metrices and regulatory frameworks to facilitate clinical translation and integration into existing healthcare infrastructures.
- Exploring multifunctional sensing platforms capable of simultaneously monitoring multiple physiological parameters (e.g., strain, temperature, glucose) under varying environmental conditions.

By addressing these challenges, NC HG-based wearable sensors have the potential to revolutionize healthcare through personalized, non-invasive, and real-time monitoring solutions.

## Figures and Tables

**Figure 4:**
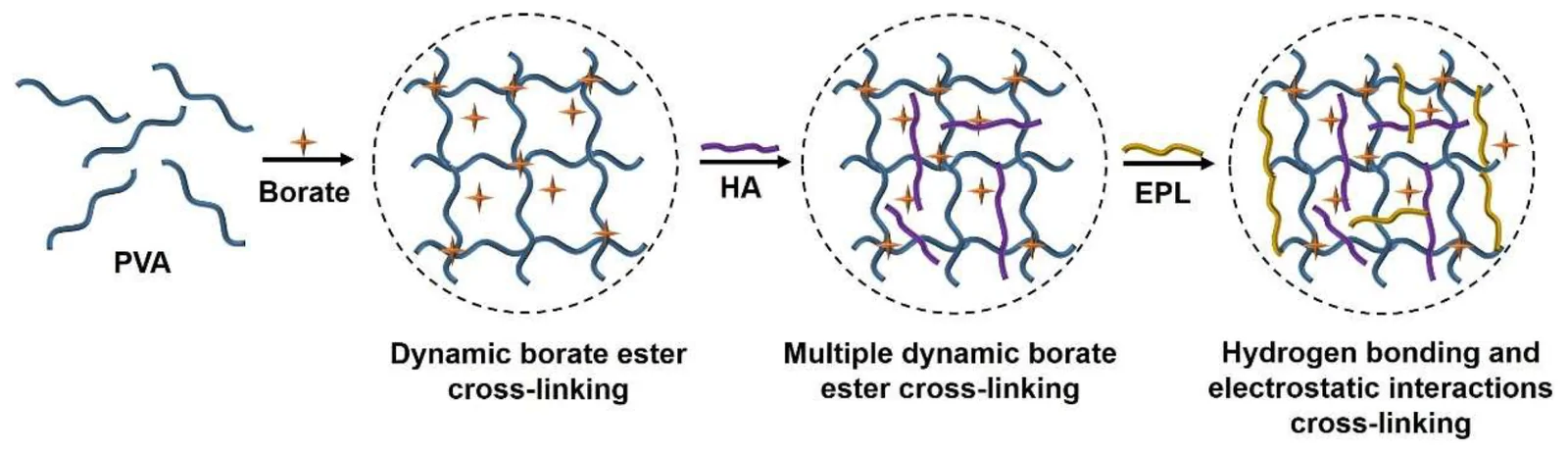
Schematic diagram of the mechanism of composite dynamic network HG through dynamic borate ester reaction and physical crosslinking. Reused from [93] under an open access license, MDPI.

**Figure 5:**
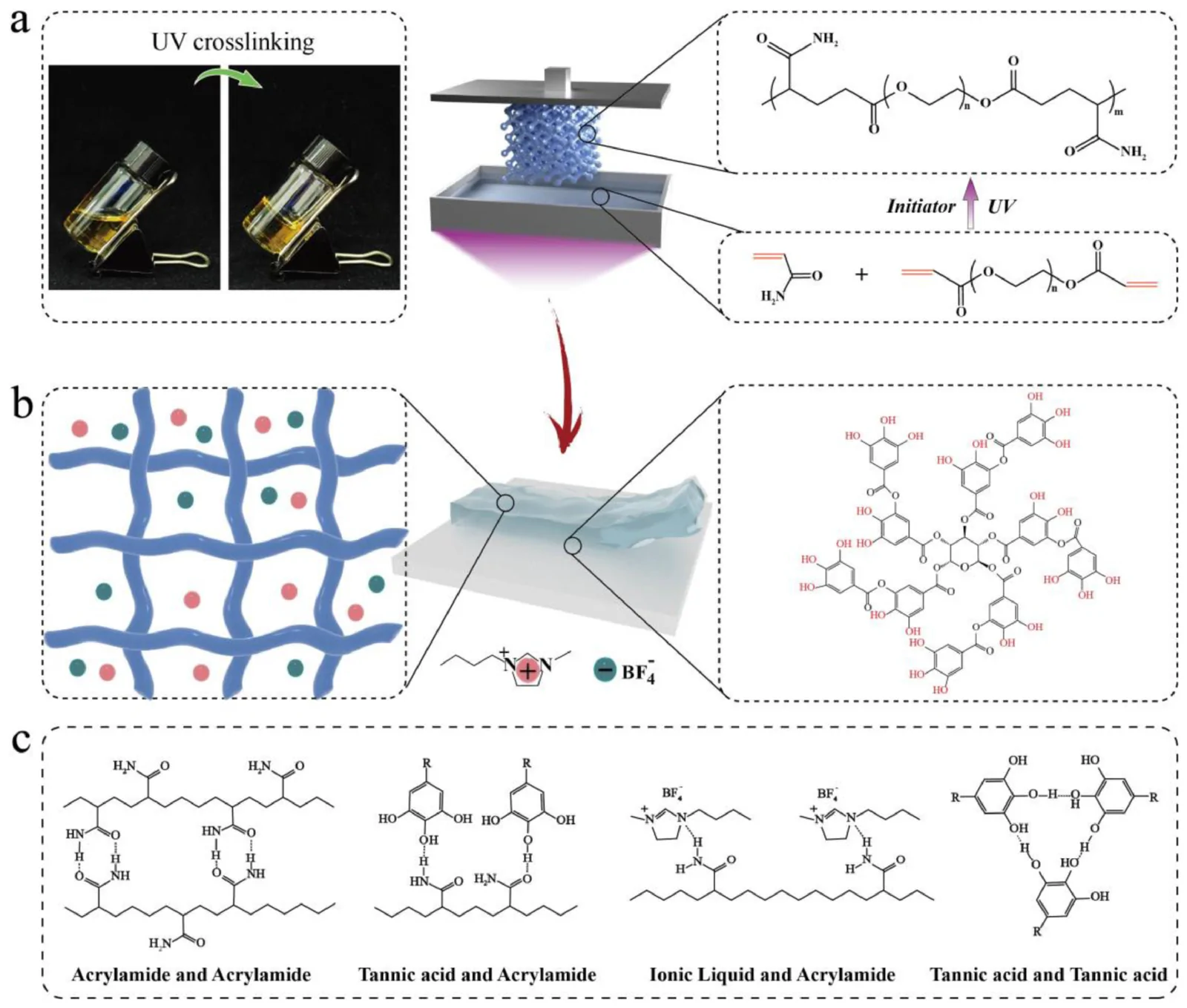
(**a**) Schematic illustration of the 3D printing and chemical crosslinking process of PAP HG; (**b**) schematic of the conductive IL component (**left**) and chemical structure of the adhesive TA component (**right**); and (**c**) predominant H-bonding interactions within the HG. Reused from [156] under an open access license, Elsevier.

**Figure 6:**
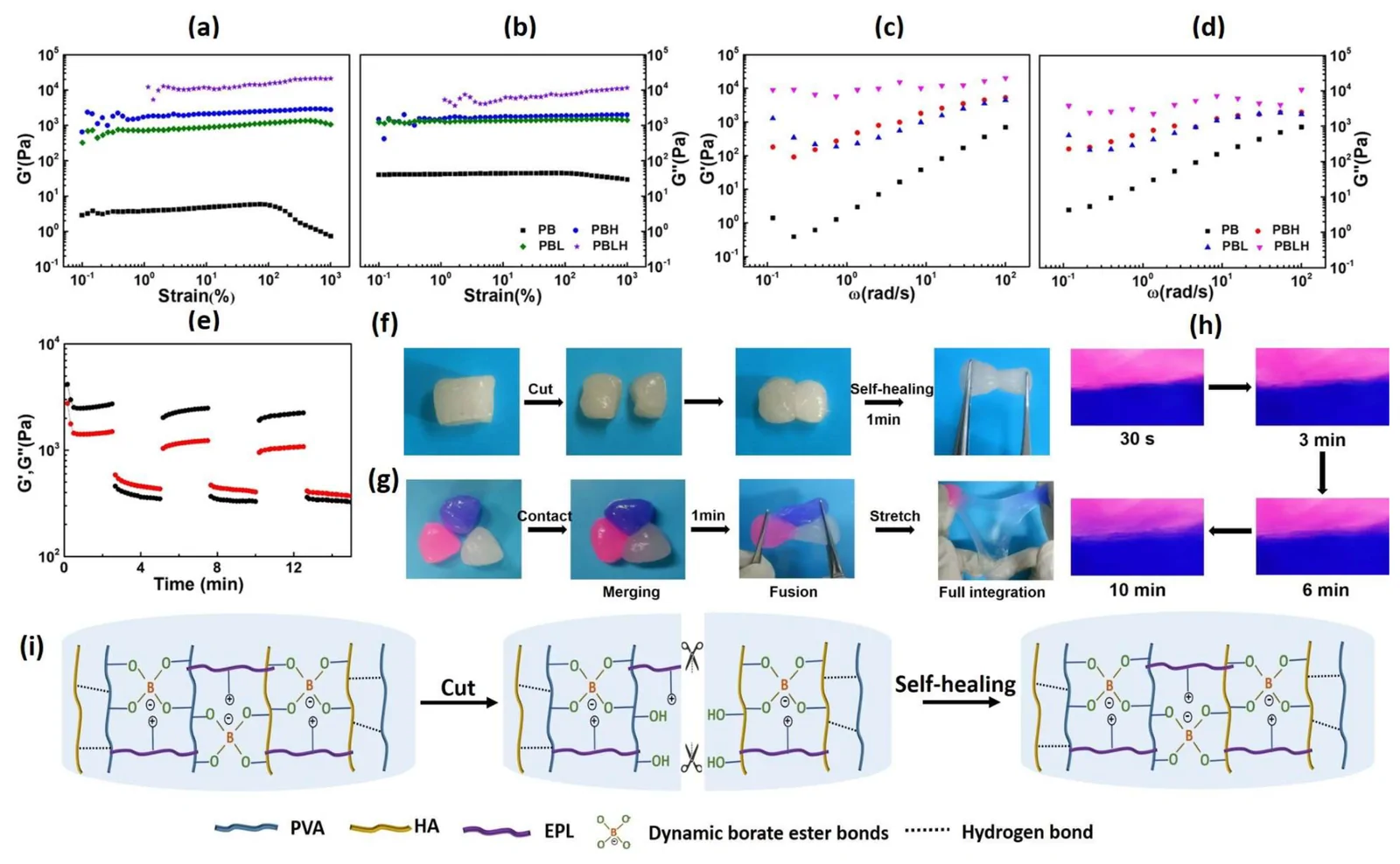
(**a**,**b**) Dynamic oscillatory strain sweep tests (from 0.1–1000%) and (**c**,**d**) frequency sweep tests (from 0.1 rad/s to 100 rad/s) of the HGs. (**e**) G’ and G″ of the PBLH HGs under alternate low (2%) and high (100%) strain at a fixed frequency of 1%; images of the in situ (**f**) (unstained HGs) and non-in situ (**g**) (stained HGs) methods were used to observe self-healing properties; the HGs were stained by methylene blue and eosin, respectively. (**h**) Photographs of the self-healing process of PBLH HGs by microscope. (**i**) Schematic diagram of the self-healing mechanism of PBLH HGs. Reused from [93] under an open access license, MDPI.

**Figure 7:**
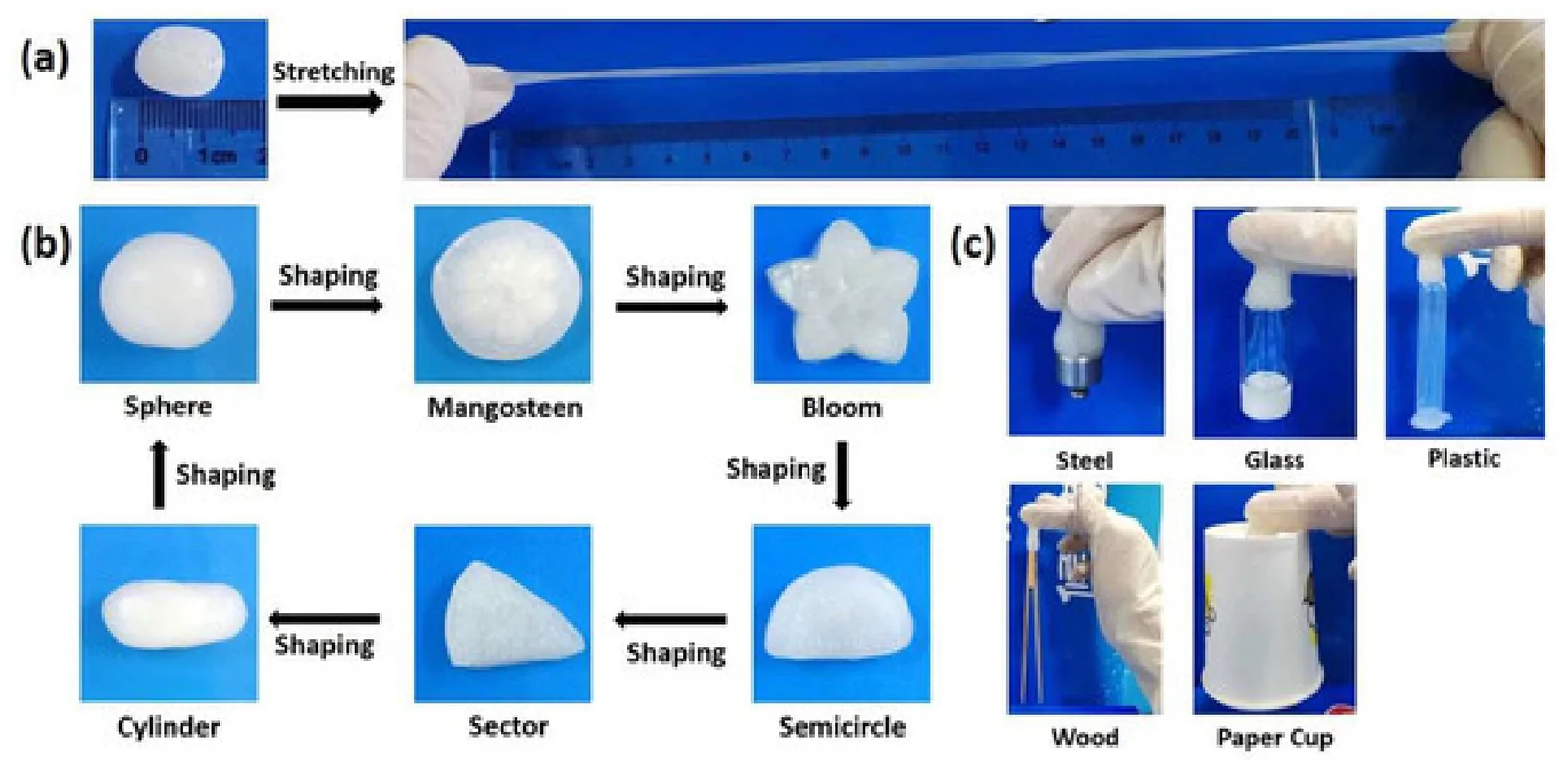
Photographs of the (**a**) stretchable, (**b**) shapeable, and (**c**) adhesive properties of the PBLH composite HGs. Reused from [93] under an open access license, MDPI.

**Figure 8:**
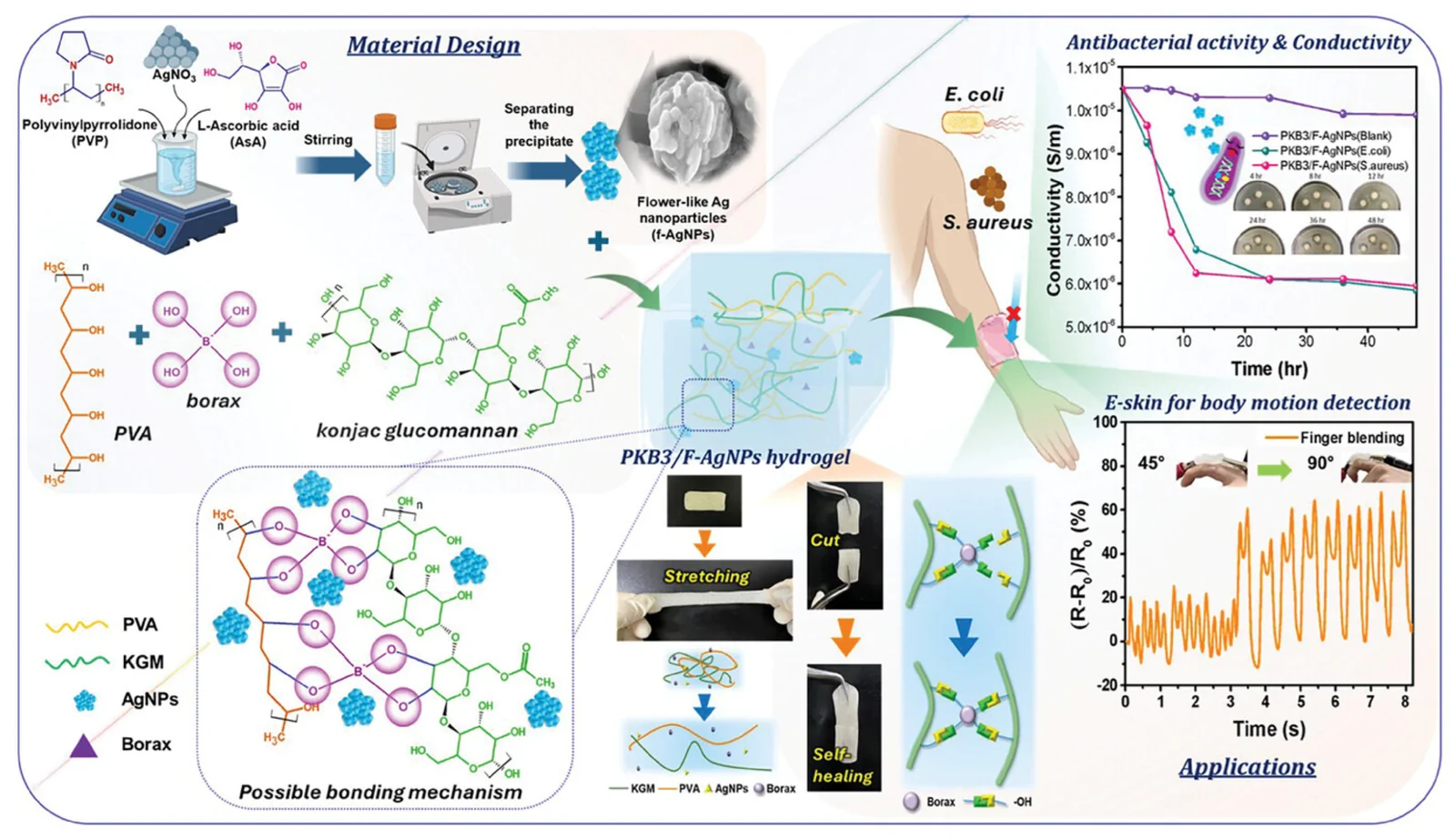
Conceptual illustration of multifunctional PKB3/F-AgNPs HGs. Reused from [200] with permission from John Wiley & Sons, Inc.

**Figure 9:**
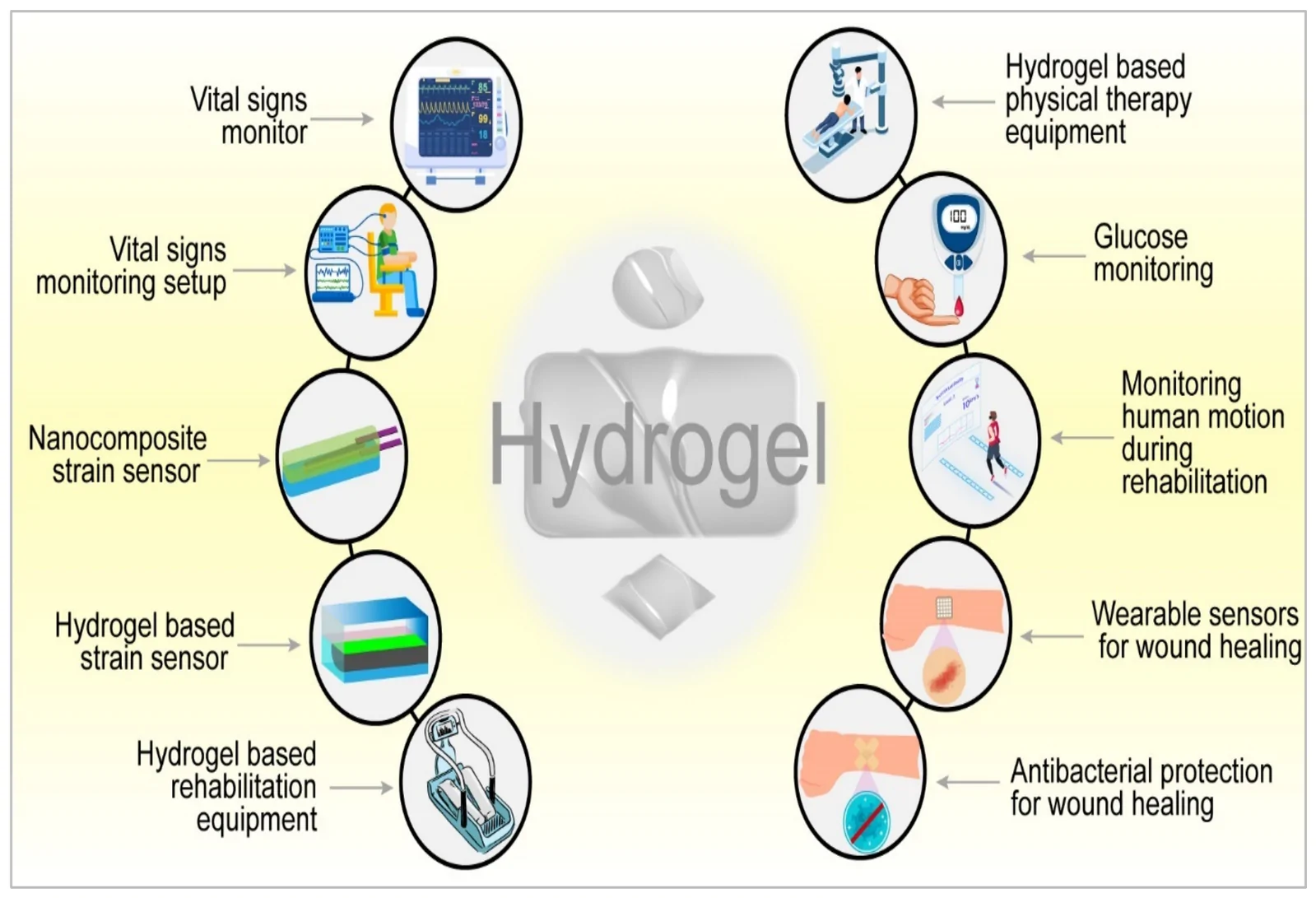
Different types of healthcare applications of NC HG sensors (created with Illustrator).

**Table 2:** Correlation of major bio-based polymers and their crosslinking methods.

Polymer	Primary Crosslinking Methods	Bond/Mechanism Type	Key Benefits	Trade-Offs & Risks	Refs.
Agarose	Temperature-induced gelation.	H-bonding (physical)	Purely physical, non-toxic; highly reversible.	Poor mechanical strength at higher temperatures; brittle structure.	[96]
Alginate	Ionic crosslinking; covalent crosslinking.	Ionic (with divalent cations like Ca^2+^); Schiff base/hydrazone (dynamic covalent)	Rapid gelation at room temperature; highly biocompatible for cell encapsulation.	Susceptible to ion exchange (degrades in saline environments); covalent adjustments require chemical modification.	[97]
Gelatin	Temperature-induced gelation; enzymatic crosslinking; NPS crosslinking.	Triple-helix formation (physical); isopeptide bonds (via transglutaminase); Imine/Schiff base bonds	Excellent cell-adhesion properties; enzymatically stable at body temperature.	Raw gelatin melts at 37 °C without secondary crosslinking; enzymatic kinetics can be hard to control.	[98]
Chitosan	Ionic crosslinking; self-crosslinking; covalent crosslinking.	Electrostatic (with polyanions like TPP); hydrophobic/hydrogen interactions; dynamic Schiff base.	Innate antimicrobial properties; pH-responsive behavior.	Soluble only in acidic conditions; chemical crosslinkers (like glutaraldehyde) can introduce cytotoxicity.	[99]
Casein	Enzymatic crosslinking; self-crosslinking.	Isopeptide bonds (via transglutaminase); micellar aggregation/hydrophobic effect.	Highly structural and biodegradable; great for food-grade and oral delivery systems.	High batch-to-batch variability; potential allergenicity.	[100]

**Table 4:** Conductivity and sensitivity of composite HGs according to various conductive materials.

Types	Conductive Materials	HG Networks	Conductivity	GF	Refs.
Liquid metal	ionic liquids	AAm-co-PEGDA	-	1–3	[156]
Metal ion	Al^3+^/PVA	PPy/CMC	3.47 S m^−1^	2.58	[163]
Conductive NMs	Ag NPs	AgNP/CNTs	-	1.755	[162]
Metal ion	Fe^3+^/Cu^2+^	DA/Py/AM	0.954 S m^−1^	7.44	[164]
Conductive NMs	Ag NPs	PVA/CNC	4.61 S m^−1^	-	[165]
Liquid metal	EGaIn	PVA/TA	3.63 S m^−1^	2.59	[166]
Conductive NMs	CNTs	PAA/SA	22.5 S m^−1^	6.29	[167]
Conductive NMs	rGO	PGPHs	2.2 S m^−1^	2.78	[168]
Conductive NMs	MXene/PEDOT: PSS	PNIPAM	11.76 S m^−1^	9.93	[169]
Conductive NMs	MXene	PAAm	0.19 S m^−1^	8.21	[170]
Conductive polymers	PEDOT-S	PVA/PAAm	0.1 S m^−1^	3.46	[171]
Conductive polymers	PPy	AC/chitosan	2.61 S m^−1^	-	[172]
Conductive NMs	Ag@PDA	PAM/BA-Ag@PDA	2.51 S m^−1^	1.86	[29]
Conductive polymers	PPy	PAM-ALG	2.16 S m^−1^	4.1	[155]
Metal ion	Zn^2+^/Al^3+^	AA/Aam	4.839 S m^−1^	-	[173]
Metal ion	Fe^3+^	PVP/TA	0.79 S m^−1^	3.61	[174]
Metal ion	LiCl	P(AM-co-AA/SA)	-	3.59	[175]

**Table 5:** Antibacterial effects and sensing functions of NC HGs.

NC HG System	Antibacterial Effects	Sensing Functions	Refs.
PVA/KGM/borax + flower-AgNPs	Strong vs. *E. coli*, *S. aureus*; enhanced by blue light	Strain sensor for macro/micro motions; conductivity change also used to read antibacterial effect	[200]
QCS-based IPN + Fe–tannic NPs	>99.9% *E. coli*, >99.5% *S. aureus*	High-GF strain sensor, fast response	[204]
Chitosan/pAAm + AgNWs + MWCNTs	Strong against *S. aureus*, *E. coli*, *B. subtilis*	High-GF strain sensor, fast response	[202]
CMC/acrylic acid + SiO-TA@Ag	Prevents bacterial colonization, >95% cell viability	Wearable motion sensor + Morse-code emergency signaling	[201]
PVA–graphene–PDA–AgNPs	“Outstanding” activity vs. Gram ± microbes	Ultrasensitive e-skin strain sensors	[208]
Guar gum/acrylic acid + MXene@AgNPs	100% inhibition of *E. coli* and *S. aureus*	Stretchable strain sensor; EMI shielding	[207]

**Table 11:** Performance comparison of representative NC HG sensors for healthcare applications.

Medical Scenario & NC HG Sensors	Target Signal	Sensing Mechanism	Target Population/Validation Model	Intended Healthcare Application	Refs
Vital-sign monitoring, SA-PBAD@PHEAA	Heartbeat, strain, temperature	Dual piezoresistive–thermoresistive sensing	Wearable/personalized healthcare; human-motion-type physiological monitoring	Electronic skin & simultaneous physiological/temperature monitoring	[219]
Pulse & respiration, Ag@PDA/(CSMA-PAM)	Pulse & respiratory motion	Piezoresistive sensing through electron transport in Ag nanostructures	Rat pulse & human breathing-pattern monitoring	Epidermal pulse & respiration monitoring	[220]
Sleep-apnea monitoring, PVA-GO/LiBr	Exhaled humidity & respiratory strain	Self-powered moisture-induced metal–air redox + resistive strain sensing	Respiratory/SAHS monitoring platform	Real-time detection of abnormal breathing & telemedicine support	[221]
Diabetes management, PBA/Pro/SF-MN	Glucose	Enzyme-free electrochemical sensing through reversible PBA–glucose interaction	Continuous glucose-monitoring application	Long-term glucose monitoring	[219]
Cancer therapy, GO-Fe_3_O_4_/PNIPAM-AAm/alginate	Tumor-responsive drug release	NIR- & magnetic-field-responsive HG actuation	MCF-7 cells, in vitro	Stimuli-responsive tumor treatment	[224]
Myocardial infarction, AT-EHBPE/HA-SH	Cardiac electrophysiological conduction	Conductive hydrogel bridging damaged myocardial tissue	Rat myocardial-infarction model	Restoration of electrical integration, improved cardiac function & reduced fibrosis	[225]
Diabetic-wound monitoring, PF127@Zn/C-G	Glucose & wound pH	Glucose-triggered acidification & fluorescence activation	Murine wound model	Monitoring diabetic wounds while promoting angiogenesis & wound closure	[223]
Rehabilitation/motion monitoring, PAM/(MXene-SF)	Human motion & weak electrophysiological signals	Strain-dependent electrical sensing	Human-motion, EMG/EOG-oriented wearable application	Rehabilitation, sign-language recognition, eye tracking & temperature monitoring	[147]
Gait rehabilitation, polyimide-based iontronic pressure sensor	Plantar pressure	Iontronic capacitive sensing	Gait & rehabilitation applications	Gait analysis, injury prevention & rehabilitation training	[229]
Continuous glucose monitoring, PBA-functionalized HG optical sensor	Glucose	Surface plasmon resonance	Diabetic-patient-oriented CGM	Continuous glucose monitoring	[230]
Continuous glucose monitoring, PVA/BTCA/β-CD/GOx/AuNPs nanofibrous HG	Interstitial-fluid glucose	Amperometric sensing	Human interstitial-fluid-oriented application	Continuous/minimally invasive glucose monitoring	[234]
Wound management, TA-siRNA@BSA dual-crosslinked HG	Wound condition	Integrated responsive wound-monitoring platform	Infected/chronic wound application	Wound monitoring, gene silencing & vascular regeneration	[236]
Wound infection monitoring, pH-responsive bacterial-nanocellulose HG	Wound pH	Colorimetric sensing	Wound-dressing application	Early-stage infection detection & healing monitoring	[235]

## Data Availability

No new data were created or analyzed in this study.

## References

[1] Chen K., Lai W., Xiao W., Li L., Huang S., Xiao X. (2023). Low-temperature adaptive dual-network mxene nanocomposite hydrogel as flexible wearable strain sensors. Micromachines.

[2] Zhu P., Zhang Y., Wu A., Feng Y., Liu Y., Niu M., Han N., Lin Y., Pan Z., Mao Y. (2026). A damping and adhesive hydrogel electrode for continuous high-fidelity dynamic electrophysiological monitoring and human–machine interaction. Nano Res..

[3] Li P., Ling Z., Liu X., Bai L., Wang W., Chen H., Yang H., Yang L., Wei D. (2023). Nanocomposite hydrogels flexible sensors with functional cellulose nanocrystals for monitoring human motion and lactate in sweat. Chem. Eng. J..

[4] Li J., Ding Q., Wang H., Wu Z., Gui X., Li C., Hu N., Tao K., Wu J. (2023). Engineering smart composite hydrogels for wearable disease monitoring. Nano-Micro Lett..

[5] Wang S., Cheng H., Yao B., He H., Zhang L., Yue S., Wang Z., Ouyang J. (2021). Self-adhesive, stretchable, biocompatible, and conductive nonvolatile eutectogels as wearable conformal strain and pressure sensors and biopotential electrodes for precise health monitoring. ACS Appl. Mater. Interfaces.

[6] Yu X., Zheng Y., Zhang H., Wang Y., Fan X., Liu T. (2021). Fast-recoverable, self-healable, and adhesive nanocomposite hydrogel consisting of hybrid nanoparticles for ultrasensitive strain and pressure sensing. Chem. Mater..

[7] Su Y., Chen C., Pan H., Yang Y., Chen G., Zhao X., Li W., Gong Q., Xie G., Zhou Y. (2021). Piezoelectric Textiles: Muscle Fibers Inspired High-Performance Piezoelectric Textiles for Wearable Physiological Monitoring (Adv. Funct. Mater. 19/2021). Adv. Funct. Mater..

[8] Paul S.J., Elizabeth I., Gupta B.K. (2021). Ultrasensitive wearable strain sensors based on a VACNT/PDMS thin film for a wide range of human motion monitoring. ACS Appl. Mater. Interfaces.

[9] Shen K., Liu Z., Xie R., Zhang Y., Yang Y., Zhao X., Zhang Y., Yang A., Cheng Y. (2023). Nanocomposite conductive hydrogels with Robust elasticity and multifunctional responsiveness for flexible sensing and wound monitoring. Mater. Horiz..

[10] Garg V., Gupta T., Rani S., Bandyopadhyay-Ghosh S., Ghosh S.B., Qiao L., Liu G. (2021). A hierarchically designed nanocomposite hydrogel with multisensory capabilities towards wearable devices for human-body motion and glucose concentration detection. Compos. Sci. Technol..

[11] Zhang Y., Liang B., Jiang Q., Li Y., Feng Y., Zhang L., Zhao Y., Xiong X. (2020). Flexible and wearable sensor based on graphene nanocomposite hydrogels. Smart Mater. Struct..

[12] Wei P., Chen T., Chen G., Liu H., Mugaanire I.T., Hou K., Zhu M. (2019). Conductive self-healing nanocomposite hydrogel skin sensors with antifreezing and thermoresponsive properties. ACS Appl. Mater. Interfaces.

[13] Fu R., Zhong X., Xiao C., Lin J., Guan Y., Tian Y., Zhou Z., Tan G., Hu H., Zhou L. (2023). A stretchable, biocompatible, and self-powered hydrogel multichannel wireless sensor system based on piezoelectric barium titanate nanoparticles for health monitoring. Nano Energy.

[14] Dong B., Yu D., Liu W. (2023). Ultrastretchable, repairable and highly sensitive xanthan collagen nanosilver hydrogel for wide temperature flexible sensing. Chem. Eng. J..

[15] Liu Y., Yu Q., Ye L., Yang L., Cui Y. (2023). A wearable, minimally-invasive, fully electrochemically-controlled feedback minisystem for diabetes management. Lab Chip.

[16] Milenković A., Otto C., Jovanov E. (2006). Wireless sensor networks for personal health monitoring: Issues and an implementation. Comput. Commun..

[17] Alfian G., Syafrudin M., Ijaz M.F., Syaekhoni M.A., Fitriyani N.L., Rhee J. (2018). A personalized healthcare monitoring system for diabetic patients by utilizing BLE-based sensors and real-time data processing. Sensors.

[18] Gao W., Emaminejad S., Nyein H.Y.Y., Challa S., Chen K., Peck A., Fahad H.M., Ota H., Shiraki H., Kiriya D. (2016). Fully integrated wearable sensor arrays for multiplexed in situ perspiration analysis. Nature.

[19] Yilmaz T., Foster R., Hao Y. (2010). Detecting vital signs with wearable wireless sensors. Sensors.

[20] Xu J., Huang H., Sun C., Yu J., Wang M., Dong T., Wang S., Chen X., Cui T., Li J. (2023). Flexible accelerated-wound-healing antibacterial hydrogel-nanofiber scaffold for intelligent wearable health monitoring. ACS Appl. Mater. Interfaces.

[21] Wang Y., Gao Y., Tang L., Guo Y., Sha B., Jiang Y. (2026). Hydrogel-based wearable and implantable biosensors in health monitoring. Biomater. Sci..

[22] Yu Y., Liang X., Ruan H., Wang T., Li Y., Wen Z. (2025). Hydrogel-based sensors for multimodal health monitoring: From material design to intelligent sensing. Nanoscale.

[23] Du Z.-P., Wang J., Zhang J.-W., Li M.-H., Yan C.-Q. (2026). Wearable bioelectronics enabled by conductive hydrogels: From materials innovation to clinical translation. Mater. Today Bio.

[24] Mo F., Lin Y., Liu Y., Zhou P., Yang J., Ji Z., Wang Y. (2025). Advances in ionic conductive hydrogels for skin sensor applications. Mater. Sci. Eng. R Rep..

[25] Zhang N., Meng Q., Xiao M., Yang C., Zhou L., Leng H., Bao C., Feng X. (2026). Recent Advances in Hydrogel—Based Wearable Devices for Sports Monitoring and Rehabilitation: Functional Applications in Training, Injury Prevention, and Recovery. Polym. Test..

[26] Huang Y., Liu X., Wang X., Zhou W., Yuan J., Yang D., Ma L., Deng X., Zhang W. (2026). Hydrogel Sensors for Human Body Detection: Principles, Materials, and Application Frontiers. ACS Appl. Polym. Mater..

[27] Wang K., Zhang J., Li H., Wu J., Wan Q., Chen T., Liu W., Peng H., Zhang H., Luo Y. (2024). Smart hydrogel sensors for health monitoring and early warning. Adv. Sens. Res..

[28] Thirumalai D., Santhamoorthy M., Kim S.-C., Lim H.-R. (2024). Conductive polymer-based hydrogels for wearable electrochemical biosensors. Gels.

[29] Shi W., Li H., Chen J., Ching Y.C., Chuah C.H., Xu C., Liu M., Zhang J., Ching K.Y., Liang Y. (2024). Stretchable, Self-Healing, and Bioactive Hydrogel with High-Functionality N, N′-bis (acryloyl) cystamine Dynamically Bonded Ag@ polydopamine Crosslinkers for Wearable Sensors. Adv. Sci..

[30] Liu K., Zhao Z., Zheng S., Liu A., Wang Y., Chen L., Miao Q. (2024). High ion-conducting PVA nanocomposite hydrogel-based wearable piezoelectric and triboelectric sensors for harsh environments. Biomacromolecules.

[31] Moghaddasi M., Oktay B., Bingol A.B., Yanikoglu R., Muslu M., Ozbolat I.T., Ustundag C.B. (2025). Conductive Nanocomposite Hydrogels for Neural Tissue Engineering: A Systematic Scoping Review of Recent Trends. Adv. Sci..

[32] Wahid F., Zhao X.-J., Jia S.-R., Bai H., Zhong C. (2020). Nanocomposite hydrogels as multifunctional systems for biomedical applications: Current state and perspectives. Compos. Part B Eng..

[33] Lin Y., Wu A., Zhang Y., Duan H., Zhu P., Mao Y. (2025). Recent progress of nanomaterials-based composite hydrogel sensors for human–machine interactions. Discov. Nano.

[34] Alshangiti D.M., El-Damhougy T.K., Zaher A., Madani M. (2023). Revolutionizing biomedicine: Advancements, applications, and prospects of nanocomposite macromolecular carbohydrate-based hydrogel biomaterials: A review. RSC Adv..

[35] Thoniyot P., Tan M.J., Karim A.A., Young D.J., Loh X.J. (2015). Nanoparticle–hydrogel composites: Concept, design, and applications of these promising, multi-functional materials. Adv. Sci..

[36] Wu X.-L., Wen T., Guo H.-L., Yang S., Wang X., Xu A.-W. (2013). Biomass-derived sponge-like carbonaceous hydrogels and aerogels for supercapacitors. ACS Nano.

[37] Xu Y., Lin Z., Huang X., Wang Y., Huang Y., Duan X. (2013). Functionalized graphene hydrogel-based high-performance supercapacitors. Adv. Mater..

[38] Wu Z.-S., Zhou G., Yin L.-C., Ren W., Li F., Cheng H.-M. (2012). Graphene/metal oxide composite electrode materials for energy storage. Nano Energy.

[39] Zhang L., Jean S.R., Ahmed S., Aldridge P.M., Li X., Fan F., Sargent E.H., Kelley S.O. (2017). Multifunctional quantum dot DNA hydrogels. Nat. Commun..

[40] Anjali J., Jose V.K., Lee J.-M. (2019). Carbon-based hydrogels: Synthesis and their recent energy applications. J. Mater. Chem. A.

[41] Zhang L.L., Zhao X. (2009). Carbon-based materials as supercapacitor electrodes. Chem. Soc. Rev..

[42] Huang Y., Liang J., Chen Y. (2012). An overview of the applications of graphene-based materials in supercapacitors. Small.

[43] Huang F., Wei W., Fan Q., Li L., Zhao M., Zhou Z. (2022). Super-stretchable and adhesive cellulose Nanofiber-reinforced conductive nanocomposite hydrogel for wearable Motion-monitoring sensor. J. Colloid Interface Sci..

[44] Salimiyan N., Gholami M., Sedghi R. (2023). Preparation of degradable, biocompatible, conductive and multifunctional chitosan/thiol-functionalized graphene nanocomposite hydrogel via click chemistry for human motion sensing. Chem. Eng. J..

[45] Zhou H., Jin Z., Gao Y., Wu P., Lai J., Li S., Jin X., Liu H., Chen W., Wu Y. (2022). Thermoresponsive, magnetic, adhesive and conductive nanocomposite hydrogels for wireless and non-contact flexible sensors. Colloids Surf. A Physicochem. Eng. Asp..

[46] Biondi M., Borzacchiello A., Mayol L., Ambrosio L. (2015). Nanoparticle-integrated hydrogels as multifunctional composite materials for biomedical applications. Gels.

[47] Sharma G., Thakur B., Naushad M., Kumar A., Stadler F.J., Alfadul S.M., Mola G.T. (2018). Applications of nanocomposite hydrogels for biomedical engineering and environmental protection. Environ. Chem. Lett..

[48] Moini N., Jahandideh A., Anderson G. (2019). Inorganic nanocomposite hydrogels: Present knowledge and future challenge. Sustainable Polymer Composites and Nanocomposites.

[49] Tipa C., Cidade M.T., Borges J.P., Costa L.C., Silva J.C., Soares P.I. (2022). Clay-based nanocomposite hydrogels for biomedical applications: A review. Nanomaterials.

[50] Wahid F., Zhong C., Wang H.-S., Hu X.-H., Chu L.-Q. (2017). Recent advances in antimicrobial hydrogels containing metal ions and metals/metal oxide nanoparticles. Polymers.

[51] Mohan Y.M., Vimala K., Thomas V., Varaprasad K., Sreedhar B., Bajpai S., Raju K.M. (2010). Controlling of silver nanoparticles structure by hydrogel networks. J. Colloid Interface Sci..

[52] Raguvaran R., Manuja B.K., Chopra M., Thakur R., Anand T., Kalia A., Manuja A. (2017). Sodium alginate and gum acacia hydrogels of ZnO nanoparticles show wound healing effect on fibroblast cells. Int. J. Biol. Macromol..

[53] Kuilla T., Bhadra S., Yao D., Kim N.H., Bose S., Lee J.H. (2010). Recent advances in graphene based polymer composites. Prog. Polym. Sci..

[54] Mohan Y.M., Lee K., Premkumar T., Geckeler K.E. (2007). Hydrogel networks as nanoreactors: A novel approach to silver nanoparticles for antibacterial applications. Polymer.

[55] Tan S., Ladewig K., Fu Q., Blencowe A., Qiao G.G. (2014). Cyclodextrin-based supramolecular assemblies and hydrogels: Recent advances and future perspectives. Macromol. Rapid Commun..

[56] Miyake T., Asakawa T. (2005). Recently developed catalytic processes with bimetallic catalysts. Appl. Catal. A Gen..

[57] Gaharwar A.K., Peppas N.A., Khademhosseini A. (2014). Nanocomposite hydrogels for biomedical applications. Biotechnol. Bioeng..

[58] Chen W., Ming Y., Wang M., Huang M., Liu H., Huang Y., Huang Z., Qing L., Wang Q., Jia B. (2023). Nanocomposite hydrogels in regenerative medicine: Applications and challenges. Macromol. Rapid Commun..

[59] Catoira M.C., Fusaro L., Di Francesco D., Ramella M., Boccafoschi F. (2019). Overview of natural hydrogels for regenerative medicine applications. J. Mater. Sci. Mater. Med..

[60] Tibbitt M.W., Anseth K.S. (2009). Hydrogels as extracellular matrix mimics for 3D cell culture. Biotechnol. Bioeng..

[61] Wanniarachchi P.C., Paranagama I.T., Idangodage P.A., Nallaperuma B., Samarasinghe T.T., Jayathilake C. (2025). Natural polymer-based hydrogels: Types, functionality, food applications, environmental significance and future perspectives: An updated review. Food Biomacromol..

[62] Santhamoorthy M., Kim S.-C. (2025). A review of the development of biopolymer hydrogel-based scaffold materials for drug delivery and tissue engineering applications. Gels.

[63] Naranjo-Alcazar R., Bendix S., Groth T., Gallego Ferrer G. (2023). Research progress in enzymatically cross-linked hydrogels as injectable systems for bioprinting and tissue engineering. Gels.

[64] Wurm F., Rietzler B., Pham T., Bechtold T. (2020). Multivalent ions as reactive crosslinkers for biopolymers—A review. Molecules.

[65] Tordi P., Tamayo A., Jeong Y., Han B., Al Kayal T., Cavallo A., Bonini M., Samorì P. (2026). Fully Bio-Based Gelatin Organohydrogels via Enzymatic Crosslinking for Sustainable Soft Strain and Temperature Sensing. Adv. Funct. Mater..

[66] Roas-Escalona N., Becquart F., Delair T., Dutertre F. (2024). Chitosan-based hydrogels: Influence of crosslinking strategy on rheological properties. Carbohydr. Polym..

[67] Takeno H., Kimura Y., Nakamura W. (2017). Mechanical, swelling, and structural properties of mechanically tough clay-sodium polyacrylate blend hydrogels. Gels.

[68] Hu Z., Ren J., Wan L., Zhang L., Yang X., Lin T. (2026). A Self-Powered and Highly Sensitive Flexible Contact-Pressure Sensor for Dynamic Sensing Based on Graphene-Enhanced Hydrogel. Nanomaterials.

[69] Chen B., Yu R., Wang J., Feng Y., Zhang Y., Mao Y., Shan C., Wang X. (2026). Biomaterials-Based Hydrogel with Superior Bio-Mimetic Ionic Conductivity and Tissue-Matching Softness for Bioelectronics. Adv. Funct. Mater..

[70] Zhang L., Huang X., Liu L., Nasar N.K.A., Gu X., Davis T.P., Zheng X., Wang L., Qiao R. (2025). Fabrication of Organic/Inorganic Nanocomposites: From Traditional Synthesis to Additive Manufacturing. Adv. Mater..

[71] Adnan M.M., Dalod A.R., Balci M.H., Glaum J., Einarsrud M.-A. (2018). In situ synthesis of hybrid inorganic–polymer nanocomposites. Polymers.

[72] Vijayakumar V., Anothumakkool B., Kurungot S., Winter M., Nair J.R. (2021). In situ polymerization process: An essential design tool for lithium polymer batteries. Energy Environ. Sci..

[73] Chen H., Ginzburg V.V., Yang J., Yang Y., Liu W., Huang Y., Du L., Chen B. (2016). Thermal conductivity of polymer-based composites: Fundamentals and applications. Prog. Polym. Sci..

[74] Schmidt B.V. (2020). Metal-organic frameworks in polymer science: Polymerization catalysis, polymerization environment, and hybrid materials. Macromol. Rapid Commun..

[75] Li Y., Yang D., Wu Z., Gao F.-L., Gao X.-Z., Zhao H.-Y., Li X., Yu Z.-Z. (2023). Self-adhesive, self-healing, biocompatible and conductive polyacrylamide nanocomposite hydrogels for reliable strain and pressure sensors. Nano Energy.

[76] Rahman M.S., Rahman M.T., Kumar H., Kim K., Kim S. (2024). ZIF-8-enhanced multifunctional, high-performance nanocomposite hydrogel–based wearable strain sensor for healthcare applications. Adv. Compos. Hybrid Mater..

[77] Hao S., Shao C., Meng L., Cui C., Xu F., Yang J. (2020). Tannic acid–silver dual catalysis induced rapid polymerization of conductive hydrogel sensors with excellent stretchability, self-adhesion, and strain-sensitivity properties. ACS Appl. Mater. Interfaces.

[78] Peng L., Su Y., Yang X., Sui G. (2023). A liquid metal/carbon nanotubes complex enabling ultra-fast polymerization of super-robust, stretchable adhesive hydrogels for highly sensitive sensor. J. Colloid Interface Sci..

[79] Yu Y., Kong K., Mu Z., Zhao Y., Liu Z., Tang R. (2020). Muscle-like ultratough hybrid hydrogel constructed by heterogeneous inorganic polymerization on an organic network. ACS Appl. Mater. Interfaces.

[80] Zou Y., Liu G., Wang H., Du K., Guo J., Shang Z., Guo R., Zhou F., Liu W. (2024). Ultra-Stretchable Composite Organohydrogels Polymerized Based on MXene@ Tannic Acid-Ag Autocatalytic System for Highly Sensitive Wearable Sensors. Small.

[81] Cui C., Shao C., Meng L., Yang J. (2019). High-strength, self-adhesive, and strain-sensitive chitosan/poly (acrylic acid) double-network nanocomposite hydrogels fabricated by salt-soaking strategy for flexible sensors. ACS Appl. Mater. Interfaces.

[82] Sun Q., Xiao L., Nie Y., Wang W., Bai L., Chen H., Yang L., Yang H., Wei D. (2022). Fabrication of Janus-type nanocomposites from cellulose nanocrystals for self-healing hydrogels’ flexible sensors. Colloids Surf. B Biointerfaces.

[83] Harun-Ur-Rashid M., Imran A.B. (2024). Emerging trends in engineering polymers: A paradigm shift in material engineering. Recent Prog. Mater..

[84] Shao Z., Hu X., Cheng W., Zhao Y., Hou J., Wu M., Xue D., Wang Y. (2020). Degradable self-adhesive epidermal sensors prepared from conductive nanocomposite hydrogel. Nanoscale.

[85] Li X., He L., Li Y., Chao M., Li M., Wan P., Zhang L. (2021). Healable, degradable, and conductive MXene nanocomposite hydrogel for multifunctional epidermal sensors. ACS Nano.

[86] Ma D., Wu X., Wang Y., Liao H., Wan P., Zhang L. (2019). Wearable, antifreezing, and healable epidermal sensor assembled from long-lasting moist conductive nanocomposite organohydrogel. ACS Appl. Mater. Interfaces.

[87] Wang W., Zhou H., Xu Z., Li Z., Zhang L., Wan P. (2024). Flexible conformally bioadhesive MXene hydrogel electronics for machine learning-facilitated human-interactive sensing. Adv. Mater..

[88] Zhang Z., Yang J., Wang H., Wang C., Gu Y., Xu Y., Lee S., Yokota T., Haick H., Someya T. (2024). A 10-micrometer-thick nanomesh-reinforced gas-permeable hydrogel skin sensor for long-term electrophysiological monitoring. Sci. Adv..

[89] Chenani H., Saeidi M., Rastkhiz M.A., Bolghanabadi N., Aghaii A.H., Orouji M., Hatamie A., Simchi A. (2024). Challenges and advances of hydrogel-based wearable electrochemical biosensors for real-time monitoring of biofluids: From lab to market. A review. Anal. Chem..

[90] Chen D., Miao Y., Xu D., Li S., Li T., Peng C., Guo X., Wu Z. (2025). Micro cross-linking strategy with dynamic covalent bond for high-performance epoxy-based thermoplastic polymer and polymer composites. Compos. Part A Appl. Sci. Manuf..

[91] Chen F., Gao F., Guo X., Liao L., Gao X., Shen L., Ma J. (2025). Enhancing polyacrylate covalent adaptable networks through dynamic protection and deprotection strategies for highly reactive functional monomers. Chem. Eng. J..

[92] Luo W., Zou M., Wang J., Ma Y., Luo L., Hu X., Chen W., Jiang X., Li Q. (2025). Cross-Linked Polyurethane Phase Change Materials Based on Multiple Hydrogen Bonds with Excellent Mechanical, Flame Retardant and Energy Storage Properties for Novel Battery Jackets. ACS Sustain. Chem. Eng..

[93] Sun N., Liu X., Lv W., Xu C., Zhang A., Sun P. (2024). Composite Hydrogels with Rapid Self-Healing, Stretchable, Moldable and Antibacterial Properties Based on PVA/ε-Poly-l-lysine/Hyaluronic Acid. Molecules.

[94] Zhao L., Zhou Y., Zhang J., Liang H., Chen X., Tan H. (2023). Natural polymer-based hydrogels: From polymer to biomedical applications. Pharmaceutics.

[95] Nanda D., Behera D., Pattnaik S.S., Behera A.K. (2025). Advances in natural polymer-based hydrogels: Synthesis, applications, and future directions in biomedical and environmental fields. Discov. Polym..

[96] Zarrintaj P., Manouchehri S., Ahmadi Z., Saeb M.R., Urbanska A.M., Kaplan D.L., Mozafari M. (2018). Agarose-based biomaterials for tissue engineering. Carbohydr. Polym..

[97] Lee K.Y., Mooney D.J. (2012). Alginate: Properties and biomedical applications. Prog. Polym. Sci..

[98] Gorgieva S., Kokol V. (2011). Collagen-vs. gelatine-based biomaterials and their biocompatibility: Review and perspectives. Biomater. Appl. Nanomed..

[99] Berger J., Reist M., Mayer J., Felt O., Peppas N., Gurny R. (2004). Structure and interactions in covalently and ionically crosslinked chitosan hydrogels for biomedical applications. Eur. J. Pharm. Biopharm..

[100] Partschefeld C., Schwarzenbolz U., Richter S., Henle T. (2007). Crosslinking of casein by microbial transglutaminase and its resulting influence on the stability of micelle structure. Biotechnol. J. Healthc. Nutr. Technol..

[101] Dai X., Huang L.-B., Du Y., Han J., Kong J. (2021). Self-healing flexible strain sensors based on dynamically cross-linked conductive nanocomposites. Compos. Commun..

[102] Xiong L., Jin S., Zhang F., Li K., Li J., Mei C., Han J., Xiao H., Seidi F. (2022). Bioinspired fabrication of self-recovery, adhesive, and flexible conductive hydrogel sensor driven by dynamic borate ester bonds and tannic acid-mediated noncovalent network. Eur. Polym. J..

[103] Fan X., Geng J., Wang Y., Gu H. (2022). PVA/gelatin/β-CD-based rapid self-healing supramolecular dual-network conductive hydrogel as bidirectional strain sensor. Polymer.

[104] Nelson B.R., Kirkpatrick B.E., Miksch C.E., Davidson M.D., Skillin N.P., Hach G.K., Khang A., Hummel S.N., Fairbanks B.D., Burdick J.A. (2024). Photoinduced dithiolane crosslinking for multiresponsive dynamic hydrogels. Adv. Mater..

[105] Wang Q., Zhang Y., Ma Y., Wang M., Pan G. (2023). Nano-crosslinked dynamic hydrogels for biomedical applications. Mater. Today Bio.

[106] Mao J., Zhao C., Li Y., Xiang D., Wang Z. (2020). Highly stretchable, self-healing, and strain-sensitive based on double-crosslinked nanocomposite hydrogel. Compos. Commun..

[107] Zeng X., Teng L., Wang X., Lu T., Leng W., Wu X., Li D., Zhong Y., Sun X., Zhu S. (2025). Efficient multi-physical crosslinked nanocomposite hydrogel for a conformal strain and self-powered tactile sensor. Nano Energy.

[108] Chen K., Hu Y., Wang F., Liu M., Liu P., Li C., Yu Y., Xiao X., Feng Q. (2022). Ultra-stretchable, adhesive, and self-healing MXene/polyampholytes hydrogel as flexible and wearable epidermal sensors. Colloids Surf. A Physicochem. Eng. Asp..

[109] Fu D., Xie Y., Zhou L., Zhang L., Zheng T., Shen J. (2024). Triple physical cross-linking cellulose nanofibers-based poly (ionic liquid) hydrogel as wearable multifunctional sensors. Carbohydr. Polym..

[110] Chang Y.-C., Liang Y.-Y., Liu H.-J. (2024). Flexible, self-healing, and degradable polymeric dielectrics cross-linked through metal–ligand for resistive memory device. Flex. Print. Electron..

[111] Roppolo I., Caprioli M., Pirri C.F., Magdassi S. (2024). 3D printing of self-healing materials. Adv. Mater..

[112] Kaliaraj G.S., Shanmugam D.K., Dasan A., Mosas K.K.A. (2023). Hydrogels—A promising materials for 3D printing technology. Gels.

[113] Bernal-Chávez S.A., Romero-Montero A., Hernández-Parra H., Peña-Corona S.I., Del Prado-Audelo M.L., Alcalá-Alcalá S., Cortés H., Kiyekbayeva L., Sharifi-Rad J., Leyva-Gómez G. (2023). Enhancing chemical and physical stability of pharmaceuticals using freeze-thaw method: Challenges and opportunities for process optimization through quality by design approach. J. Biol. Eng..

[114] Adelnia H., Ensandoost R., Moonshi S.S., Gavgani J.N., Vasafi E.I., Ta H.T. (2022). Freeze/thawed polyvinyl alcohol hydrogels: Present, past and future. Eur. Polym. J..

[115] Wang P., Li G., Liu J., Hou Z., Meng C., Guo S., Liu C., Fan S. (2021). Tailorable capacitive tactile sensor based on stretchable and dissolvable porous silver nanowire/polyvinyl alcohol nanocomposite hydrogel for wearable human motion detection. Adv. Mater. Interfaces.

[116] Hu J., Wu Y., Yang Q., Zhou Q., Hui L., Liu Z., Xu F., Ding D. (2022). One-pot freezing-thawing preparation of cellulose nanofibrils reinforced polyvinyl alcohol based ionic hydrogel strain sensor for human motion monitoring. Carbohydr. Polym..

[117] Zhang W., Wen J.-Y., Ma M.-G., Li M.-F., Peng F., Bian J. (2021). Anti-freezing, water-retaining, conductive, and strain-sensitive hemicellulose/polypyrrole composite hydrogels for flexible sensors. J. Mater. Res. Technol..

[118] Zhang M., Wang Y., Liu K., Liu Y., Xu T., Du H., Si C. (2023). Strong, conductive, and freezing-tolerant polyacrylamide/PEDOT: PSS/cellulose nanofibrils hydrogels for wearable strain sensors. Carbohydr. Polym..

[119] Nie Y., Yue D., Xiao W., Wang W., Chen H., Bai L., Yang L., Yang H., Wei D. (2022). Anti-freezing and self-healing nanocomposite hydrogels based on poly (vinyl alcohol) for highly sensitive and durable flexible sensors. Chem. Eng. J..

[120] Wu W., Shi L., Qian K., Zhou J., Zhao T., Thaiboonrod S., Miao M., Feng X. (2024). Synergistic strengthening of PVA ionic conductive hydrogels using aramid nanofibers and tannic acid for mechanically robust, antifreezing, water-retaining and antibacterial flexible sensors. J. Colloid Interface Sci..

[121] Takeno H., Narita T., Hsieh W.-C., Saito K., Ku Y.-T., Su Y.-C., Inoguchi H. (2024). Effects of repeated freeze-thaw cycles on the mechanical and structural properties of cellulose nanofiber and poly (vinyl alcohol) hydrogels. Cellulose.

[122] Monta K., Hidaka M., Ishikawa D., Fujii T. (2024). State analysis of a biobased hydrogel subjected to freeze and thaw processes by X ray absorption spectroscopy using synchrotron radiation. Sci. Rep..

[123] Lu Y., Yue Y., Ding Q., Mei C., Xu X., Wu Q., Xiao H., Han J. (2021). Self-recovery, fatigue-resistant, and multifunctional sensor assembled by a nanocellulose/carbon nanotube nanocomplex-mediated hydrogel. ACS Appl. Mater. Interfaces.

[124] Feng Y., Yu J., Sun D., Ren W., Shao C., Sun R. (2022). Solvent-induced in-situ self-assembly lignin nanoparticles to reinforce conductive nanocomposite organogels as anti-freezing and anti-dehydration flexible strain sensors. Chem. Eng. J..

[125] Liu X., Ma Y., Zhang X., Huang J. (2021). Cellulose nanocrystal reinforced conductive nanocomposite hydrogel with fast self-healing and self-adhesive properties for human motion sensing. Colloids Surf. A Physicochem. Eng. Asp..

[126] Yang X., Li C., Xia J., Zhang F., Wang Z. (2024). Self-assembly of a AuNPs/Ti3C2 MXene hydrogel for cascade amplification of microRNA-122 biosensing. Microchim. Acta.

[127] Wang J., Du P., Hsu Y.-I., Uyama H. (2024). Rapid preparation of dynamic-crosslinked nanocomposite hydrogel sensors with efficiency self-healing and adhesion properties for elderly health and sleep management. Chem. Eng. J..

[128] Liang X., Zhang M., Chong C.-M., Lin D., Chen S., Zhen Y., Ding H., Zhong H.-J. (2024). Recent advances in the 3D printing of conductive hydrogels for sensor applications: A review. Polymers.

[129] Teekayupak K., Preechakasedkit P., Chuaypen N., Dissayabutra T., Lieberzeit P.A., Chailapakul O., Ruecha N., Citterio D. (2025). Polymeric hydrogel integrated paper-based potentiometric ion-sensing device for the determination of sodium ions in human urine. Analyst.

[130] Jiang W., Liu J., Zhang H., Song D., Yu J., Liu Q., Chen R., Zhu J., Wang J. (2024). Low-temperature resistant hydrogel with inkjet-printed MXene on microspine surface for pressure sensing and triboelectric energy harvesting. Chem. Eng. J..

[131] Huang B., Zhao Z., Zheng Y., Xu K., Wang D., Yang Q., Yang T., Yang X., Chen H. (2024). 2D/3D-printed PEDOT/PSS conductive hydrogel for biomedical sensors. Int. J. Bioprint..

[132] Lai P.-C., Yu S.-S. (2021). Cationic cellulose nanocrystals-based nanocomposite hydrogels: Achieving 3D printable capacitive sensors with high transparency and mechanical strength. Polymers.

[133] Zhou P., Liao Y., Li Y., Pan D., Cao W., Yang X., Zou F., Zhou L.-m., Zhang Z., Su Z. (2019). An inkjet-printed, flexible, ultra-broadband nanocomposite film sensor for in-situ acquisition of high-frequency dynamic strains. Compos. Part A Appl. Sci. Manuf..

[134] Wang X., Zhang M., Zhang L., Xu J., Xiao X., Zhang X. (2022). Inkjet-printed flexible sensors: From function materials, manufacture process, and applications perspective. Mater. Today Commun..

[135] Cho Y., Kim K., Kim D., Bissannagari M., Lee J., Hong W., Kwon H.-J., Jang J.E., Kang H. (2024). Stretchable substrate surface-embedded inkjet-printed strain sensors for design customizable on-skin healthcare electronics. ACS Appl. Electron. Mater..

[136] Yan K., Li J., Pan L., Shi Y. (2020). Inkjet printing for flexible and wearable electronics. Apl Mater..

[137] Dutta S.D., Ganguly K., Randhawa A., Patil T.V., Kim H., Acharya R., Lim K.T. (2024). 3D Printable Hydrogel Bioelectronic Interfaces for Healthcare Monitoring and Disease Diagnosis: Materials, Design Strategies, and Applications. Adv. Mater. Technol..

[138] Li M., Chen D., Sun X., Xu Z., Yang Y., Song Y., Jiang F. (2022). An environmentally tolerant, highly stable, cellulose nanofiber-reinforced, conductive hydrogel multifunctional sensor. Carbohydr. Polym..

[139] Wei J., Liu C., Shi L., Liu Y., Lu H. (2024). One-Pot Synthesis of Organic–Inorganic Network Hydrogels with High Extensibility, Self-Healing, Self-Adhesive, 3D-Printing, and Antibacterial Properties. ACS Appl. Polym. Mater..

[140] Chen X., Song Z., Li S., Thang N.T., Gao X., Gong X., Guo M. (2020). Facile one-pot synthesis of self-assembled nitrogen-doped carbon dots/cellulose nanofibril hydrogel with enhanced fluorescence and mechanical properties. Green Chem..

[141] Sadak O. (2021). One-pot scalable synthesis of rGO/AuNPs nanocomposite and its application in enzymatic glucose biosensor. Nanocomposites.

[142] Roy A., Zenker S., Jain S., Afshari R., Oz Y., Zheng Y., Annabi N. (2024). A highly stretchable, conductive, and transparent bioadhesive hydrogel as a flexible sensor for enhanced real-time human health monitoring. Adv. Mater..

[143] Sun Z., Li Y., Zhang G., Chen Z., Ren F., Jin Y., Ren P. (2024). Recyclable Organic Ionic Conductive Hydrogels for Flexible Wearable Sensors with Excellent Environmental Resistance. ACS Appl. Electron. Mater..

[144] Xu K., Wang L., Shan W., Gao K., Wang J., Zhong Q., Zhou W. (2024). Highly stretchable and self-adhesive wearable biosensor based on nanozyme-catalyzed conductive hydrogels. ACS Appl. Polym. Mater..

[145] Yadollahi M., Farhoudian S., Namazi H. (2015). One-pot synthesis of antibacterial chitosan/silver bio-nanocomposite hydrogel beads as drug delivery systems. Int. J. Biol. Macromol..

[146] Dang X., Guo B., Fu Y., Fei Y., Wang X. (2025). All-natural biomass-based multifunctional conductive hydrogel for an all-in-one wearable strain sensor. Cell Biomater..

[147] Lu N., Kang H., Lu Y., Li Y., Li J., Xue Y., Qiu H. (2025). High-Strength, conductive dual-network nanocomposite hydrogel for multi-substrate adhesion and enhanced wearable sensor performance. Polymer.

[148] Xia W., Yu Y., Zhou C., Wang W., Wu Z., Chen H. (2023). Itaconic acid-enhanced robust ionic conductive elastomers for strain/pressure sensors. J. Mater. Chem. C.

[149] Imani K.B.C., Dodda J.M., Yoon J., Torres F.G., Imran A.B., Deen G.R., Al-Ansari R. (2024). Seamless integration of conducting hydrogels in daily life: From preparation to wearable application. Adv. Sci..

[150] Xing W., Tang Y. (2022). On mechanical properties of nanocomposite hydrogels: Searching for superior properties. Nano Mater. Sci..

[151] Zhu J., Tao J., Yan W., Song W. (2023). Pathways toward wearable and high-performance sensors based on hydrogels: Toughening networks and conductive networks. Natl. Sci. Rev..

[152] Xu A., Sun T., Liu R., Li L., Gong Y., Xiao Z. (2024). Robust hydrogel sensor with good mechanical properties, conductivity, anti-swelling ability, water tolerance and biocompatibility. Green Chem..

[153] Hui Y., Liu R., Sun T., Li L., Gong Y., Xiao Z., Xu A., Wei X. (2024). Engineering robust conductive hydrogel sensors with enhanced mechanical properties and practical applicability. Macromolecules.

[154] Liu D., Zhou H., Zhao Y., Huyan C., Wang Z., Torun H., Guo Z., Dai S., Xu B.B., Chen F. (2022). A strand entangled supramolecular PANI/PAA hydrogel enabled ultra-stretchable strain sensor. Small.

[155] Sun Z., Dong C., Chen B., Li W., Hu H., Zhou J., Li C., Huang Z. (2023). Strong, tough, and anti-swelling supramolecular conductive hydrogels for amphibious motion sensors. Small.

[156] Xu X., Jiang P., Liu D., Lyu Y., Shi X., Ji Z., Wang X. (2024). 3D printable ionic conductive hydrogels with super stretch and self-adhesion performances for flexible sensors. Giant.

[157] Liu C., Zhang R., Li P., Qu J., Chao P., Mo Z., Yang T., Qing N., Tang L. (2022). Conductive hydrogels with ultrastretchability and adhesiveness for flame-and cold-tolerant strain sensors. ACS Appl. Mater. Interfaces.

[158] Wang S., Xiang J., Sun Y., Wang H., Du X., Cheng X., Du Z., Wang H. (2021). Skin-inspired nanofibrillated cellulose-reinforced hydrogels with high mechanical strength, long-term antibacterial, and self-recovery ability for wearable strain/pressure sensors. Carbohydr. Polym..

[159] Sethi S., Medha, Thakur S., Singh A., Kaith B.S. (2022). Nanogels and nanocomposite hydrogels for sensing applications. Handbook of Green and Sustainable Nanotechnology: Fundamentals, Developments and Applications.

[160] Ahmadian Z., Kazeminava F., Afrouz M., Abbaszadeh M., Mehr N.T., Shiran J.A., Gouda C., Adeli M., Kafil H.S. (2023). A review on the impacts of metal/metal nanoparticles on characteristics of hydrogels: Special focus on carbohydrate polymers. Int. J. Biol. Macromol..

[161] Wang L., Daoud W.A. (2019). Hybrid conductive hydrogels for washable human motion energy harvester and self-powered temperature-stress dual sensor. Nano Energy.

[162] Yu X., Qin W., Li X., Wang Y., Gu C., Chen J., Yin S. (2022). Highly sensitive, weatherability strain and temperature sensors based on AgNPs@ CNT composite polyvinyl hydrogel. J. Mater. Chem. A.

[163] Zhang S., Zhu Y., Ma Z., Zhou Y., Li X., Jiang W., Xue Y., Wu X., Chen W., Qin Y.X. (2024). Constructing Electronic/Ionic-Conductive Hydrogel with Soft Compliance and High Conductivity as Flexible Strain Sensor. Adv. Eng. Mater..

[164] Mirzaei G., Mirzaei A., Javanshir S. (2024). Flexible mussel-inspired hydrogel for transparent wearable strain sensors: Investigation of mechanical, physical properties, self-healing, and electrical conductivity. J. Appl. Polym. Sci..

[165] Lin F., Wang Z., Shen Y., Tang L., Zhang P., Wang Y., Chen Y., Huang B., Lu B. (2019). Natural skin-inspired versatile cellulose biomimetic hydrogels. J. Mater. Chem. A.

[166] Zhou Z., Qian C., Yuan W. (2021). Self-healing, anti-freezing, adhesive and remoldable hydrogel sensor with ion-liquid metal dual conductivity for biomimetic skin. Compos. Sci. Technol..

[167] Wei J., Xie J., Zhang P., Zou Z., Ping H., Wang W., Xie H., Shen J.Z., Lei L., Fu Z. (2021). Bioinspired 3D printable, self-healable, and stretchable hydrogels with multiple conductivities for skin-like wearable strain sensors. ACS Appl. Mater. Interfaces.

[168] Ke X., Mu X., Chen S., Zhang Z., Zhou J., Chen Y., Gao J., Liu J., Wang X., Ma C. (2023). Reduced graphene oxide reinforced PDA-Gly-PVA composite hydrogel as strain sensors for monitoring human motion. Soft Sci..

[169] Xue P., Valenzuela C., Ma S., Zhang X., Ma J., Chen Y., Xu X., Wang L. (2023). Highly conductive MXene/PEDOT: PSS-integrated poly (N-Isopropylacrylamide) hydrogels for bioinspired somatosensory soft actuators. Adv. Funct. Mater..

[170] Wei Y., Xiang L., Ou H., Li F., Zhang Y., Qian Y., Hao L., Diao J., Zhang M., Zhu P. (2020). MXene-based conductive organohydrogels with long-term environmental stability and multifunctionality. Adv. Funct. Mater..

[171] Ji H., He H., Sun J., Lu W., Yu H., Zhu J., Liu Y., Chen S., Cao H. (2024). Sensitive wearable strain sensor based on a self-doped conductive hydrogel. ACS Appl. Electron. Mater..

[172] Zhao L., Li X., Li Y., Wang X., Yang W., Ren J. (2021). Polypyrrole-doped conductive self-healing composite hydrogels with high toughness and stretchability. Biomacromolecules.

[173] Zhang Y., Lin X., Wang Z., Zhang L., Wang S., Huang Z., Liu H., Xu X. (2024). Multiple hydrogen bonds enable high strength and anti-swelling cellulose-based ionic conductive hydrogels for flexible sensors. Chem. Eng. J..

[174] Pang Q., Hu H., Zhang H., Qiao B., Ma L. (2022). Temperature-responsive ionic conductive hydrogel for strain and temperature sensors. ACS Appl. Mater. Interfaces.

[175] Cai H., Zhang D., Zhang H., Tang M., Xu Z., Xia H., Li K., Wang J. (2023). Trehalose-enhanced ionic conductive hydrogels with extreme stretchability, self-adhesive and anti-freezing abilities for both flexible strain sensor and all-solid-state supercapacitor. Chem. Eng. J..

[176] Huang X., Wang L., Shen Z., Ren J., Chen G., Li Q., Zhou Z. (2022). Super-stretchable and self-healing hydrogel with a three-dimensional silver nanowires network structure for wearable sensor and electromagnetic interference shielding. Chem. Eng. J..

[177] Ren X., Yang M., Yang T., Xu C., Ye Y., Wu X., Zheng X., Wang B., Wan Y., Luo Z. (2021). Highly conductive PPy–PEDOT: PSS hybrid hydrogel with superior biocompatibility for bioelectronics application. ACS Appl. Mater. Interfaces.

[178] Gao Z., Sun S., Guan X., Yang Y., Sun J., Ren H., Jin Z. (2024). Ultra-high stretchability and wide temperature range adhesion hydrogels for flexible sensor. J. Appl. Polym. Sci..

[179] Huang Y., Liu B., Zhang W., Qu G., Jin S., Li X., Nie Z., Zhou H. (2022). Highly sensitive active-powering pressure sensor enabled by integration of double-rough surface hydrogel and flexible batteries. npj Flex. Electron..

[180] Li G., Li C., Li G., Yu D., Song Z., Wang H., Liu X., Liu H., Liu W. (2022). Development of conductive hydrogels for fabricating flexible strain sensors. Small.

[181] Zhu T., Ni Y., Biesold G.M., Cheng Y., Ge M., Li H., Huang J., Lin Z., Lai Y. (2023). Recent advances in conductive hydrogels: Classifications, properties, and applications. Chem. Soc. Rev..

[182] Deng Z., Wang H., Ma P.X., Guo B. (2020). Self-healing conductive hydrogels: Preparation, properties and applications. Nanoscale.

[183] Fan L., Ge X., Qian Y., Wei M., Zhang Z., Yuan W.-E., Ouyang Y. (2020). Advances in synthesis and applications of self-healing hydrogels. Front. Bioeng. Biotechnol..

[184] Huang S., Kong X., Xiong Y., Zhang X., Chen H., Jiang W., Niu Y., Xu W., Ren C. (2020). An overview of dynamic covalent bonds in polymer material and their applications. Eur. Polym. J..

[185] Guo R., Su Q., Zhang J., Dong A., Lin C., Zhang J. (2017). Facile access to multisensitive and self-healing hydrogels with reversible and dynamic boronic ester and disulfide linkages. Biomacromolecules.

[186] Zhang C., Wang M., Jiang C., Zhu P., Sun B., Gao Q., Gao C., Liu R. (2022). Highly adhesive and self-healing γ-PGA/PEDOT: PSS conductive hydrogels enabled by multiple hydrogen bonding for wearable electronics. Nano Energy.

[187] Zheng Y., Baidya A., Annabi N. (2023). Molecular design of an ultra-strong tissue adhesive hydrogel with tunable multifunctionality. Bioact. Mater..

[188] Zong S., Lv H., Liu C., Zhu L., Duan J., Jiang J. (2023). Mussel inspired Cu-tannic autocatalytic strategy for rapid self-polymerization of conductive and adhesive hydrogel sensors with extreme environmental tolerance. Chem. Eng. J..

[189] Fu Q., Hao S., Meng L., Xu F., Yang J. (2021). Engineering self-adhesive polyzwitterionic hydrogel electrolytes for flexible zinc-ion hybrid capacitors with superior low-temperature adaptability. ACS Nano.

[190] Yan Q., Zhou M., Fu H. (2020). Study on mussel-inspired tough TA/PANI@ CNCs nanocomposite hydrogels with superior self-healing and self-adhesive properties for strain sensors. Compos. Part B Eng..

[191] Bai Y., Yan S., Wang Y., Wang Q., Duan X. (2023). Facile preparation of a self-adhesive conductive hydrogel with long-term usability. ACS Appl. Mater. Interfaces.

[192] Xu Z., Bi F., Dong L., Li Z., Kuai Y. (2024). Construction of high-strength, super-adhesive conductive hydrogel based on dopamine-modified carboxymethyl cellulose under the multi-effect of iron ions. Eur. Polym. J..

[193] Ding Q., Zhou Z., Wang H., Wu Z., Tao K., Yang B.R., Xie X., Fu J., Wu J. (2023). Self-healable, recyclable, ultrastretchable, and high-performance NO2 sensors based on an organohydrogel for room and sub-zero temperature and wireless operation. SmartMat.

[194] Ma Y., Zhang D., Wang Z., Zhang H., Xia H., Mao R., Cai H., Luan H. (2023). Self-adhesive, anti-freezing MXene-based hydrogel strain sensor for motion monitoring and handwriting recognition with deep learning. ACS Appl. Mater. Interfaces.

[195] Gun’ko V.M., Savina I.N., Mikhalovsky S.V. (2017). Properties of water bound in hydrogels. Gels.

[196] Wen J., Tang J., Ning H., Hu N., Zhu Y., Gong Y., Xu C., Zhao Q., Jiang X., Hu X. (2021). Multifunctional ionic skin with sensing, UV-filtering, water-retaining, and anti-freezing capabilities. Adv. Funct. Mater..

[197] Bao S., Gao J., Xu T., Li N., Chen W., Lu W. (2021). Anti-freezing and antibacterial conductive organohydrogel co-reinforced by 1D silk nanofibers and 2D graphitic carbon nitride nanosheets as flexible sensor. Chem. Eng. J..

[198] Liu H., Zhang X.-F., Li M., Yao J. (2024). Attapulgite-Reinforced Cellulose Hydrogels with High Conductivity and Antifreezing Property for Flexible Sensors. Langmuir.

[199] Liu P., Xiao W., Bai L., Wang W., Chen H., Yang L., Yin K., Yang H., Wei D. (2024). Surface functionalization of cellulose nanocrystals for fabricating of anti-freezing and self-healing nanocomposites hydrogels. Eur. Polym. J..

[200] Cheng H., Keerthika Devi R., Huang K.Y., Ganesan M., Ravi S.K., Lin C.C. (2024). Highly biocompatible antibacterial hydrogel for wearable sensing of macro and microscale human body motions. Small.

[201] Jiang W., Peng Y., Yao J., Li Z., Wang L., Liu W. (2025). Cellulose and gum arabic based nanocomposite hydrogel sensors for health monitoring and emergency medical signaling. Int. J. Biol. Macromol..

[202] Lv A., Lv X., Tian S., Xie T., Xu X., Sun S. (2023). Tough, self-healing, and antimicrobial hydrogel sensors based on hydrogen-bonded, cross-linked chitosan and MWCNTs. ACS Appl. Polym. Mater..

[203] Abou El Fadl F.I., Hegazy D.E., Maziad N.A., Ghobashy M.M. (2023). Effect of nano-metal oxides (TiO_2_, MgO, CaO, and ZnO) on antibacterial property of (PEO/PEC-co-AAm) hydrogel synthesized by gamma irradiation. Int. J. Biol. Macromol..

[204] Cao Y., Wei Y., Feng C., Xu S., Zhang D., Dong J., Han S., Huo P. (2026). Hydrogels With Nanostructured and Antimicrobial Properties Prepared by the One-Pot Method Performance for Potential Use as Wearable Sensors. Adv. Mater. Technol..

[205] Solaiman, Foyez T., Monim S.A., Rahman A., Imran A.B. (2025). Facile synthesis of bioactive silver nanocomposite hydrogels with electro-conductive and wound-healing properties. Gels.

[206] Suneetha M., Alshehri S.A., Han S.S. (2025). Poly (dopamine) nanoparticles-reinforced bacterial cellulose-fungal carboxymethyl chitosan nanocomposite hydrogel for wearable strain sensing. Colloids Surf. A Physicochem. Eng. Asp..

[207] Pu T., Li C., Yang L., Tang X.-z., Ruan Y., Guo T. (2025). A highly stretchable, self-adhesive, antimicrobial conductive hydrogel with guar gum/acrylic acid/MXene@ AgNPs for multifunctional wearable sensors and electromagnetic interference shielding. RSC Adv..

[208] Fan L., Xie J., Zheng Y., Wei D., Yao D., Zhang J., Zhang T. (2020). Antibacterial, self-adhesive, recyclable, and tough conductive composite hydrogels for ultrasensitive strain sensing. ACS Appl. Mater. Interfaces.

[209] Liang X., Chen S., Liang Y., Wang M., Wang Q., Chen D., Ma X., Ding H., Zhong H.-J. (2026). Alginate-based hydrogels: Recent progress in preparation, property tuning, and multifunctional applications. Gels.

[210] Agyemang P., Mandal S., Hwang S. (2026). Sustainability of biodegradable superabsorbent hydrogels in agriculture. A review: P. Agyemang et al. Agron. Sustain. Dev..

[211] Gowthaman N., Lim H., Sreeraj T., Amalraj A., Gopi S. (2021). Advantages of biopolymers over synthetic polymers: Social, economic, and environmental aspects. Biopolymers and Their Industrial Applications.

[212] Fiandra E.F., Shaw L., Starck M., McGurk C.J., Mahon C.S. (2023). Designing biodegradable alternatives to commodity polymers. Chem. Soc. Rev..

[213] Segneanu A.-E., Bejenaru L.E., Bejenaru C., Blendea A., Mogoşanu G.D., Biţă A., Boia E.R. (2025). Advancements in hydrogels: A comprehensive review of natural and synthetic innovations for biomedical applications. Polymers.

[214] Ligarda-Samanez C.A., Huamán-Carrión M.L., Palomino-Rincón H., Taipe-Pardo F., Moscoso-Moscoso E., Cabel-Moscoso D.J., Garcia-Espinoza A.J., Huamaní D.F.C., Plasencia J.M.c.R., Martinez-Hernandez J.A. (2026). Sustainable biopolymers for environmental applications: Advances and future perspectives toward a circular economy. Polymers.

[215] Lam E., Martinez I., Voutchkova-Kostal A., Patel A., Wang J., Reich J., Ponnusamy E., Goetz R.J., Jin Y., Wagner M.S. (2026). Natural Polymers: Opportunities and Challenges in the Application and Commercialization as Sustainable Materials Amid Growing Environmental Awareness. ACS Sustain. Chem. Eng..

[216] Roy A., Afshari R., Jain S., Zheng Y., Lin M.-H., Zenkar S., Yin J., Chen J., Peppas N.A., Annabi N. (2025). Advances in conducting nanocomposite hydrogels for wearable biomonitoring. Chem. Soc. Rev..

[217] Kim J., Campbell A.S., de Ávila B.E.-F., Wang J. (2019). Wearable biosensors for healthcare monitoring. Nat. Biotechnol..

[218] Zhu P., Peng H., Rwei A.Y. (2022). Flexible, wearable biosensors for digital health. Med. Nov. Technol. Devices.

[219] Liu C., Li Y., Yu X. (2025). Multifunctional conductive hydrogel sensors for multiscale motion detection and wide-range temperature monitoring. ACS Appl. Polym. Mater..

[220] Wu G., Shi W., Liu M., Liang L., Wang T., Zhang J., Chen J., Liang Y., Tang W., Li H. (2025). Multifunctional strain/pressure sensor based on Ag@ polydopamine nanohybrid methacrylamide chitosan/polyacrylamide hydrogel for healthcare monitoring. ACS Appl. Mater. Interfaces.

[221] Xu Y., Yu Y., Lin N., Huang L., Zhang X., Heydari E., Bai G. (2025). Self-Powered Dual-Mode Hydrogel Sensors for Respiratory Monitoring via Humidity Sensing and Strain Detection. ACS Appl. Electron. Mater..

[222] Du K., Liu G., Zou Y., Chen Z., Guo J., Zhao N., Guo R., Zhou F., Liu W. (2025). Nanocomposite Multi-Cross-Linked Hydrogels with High Strength, High Stretchability and High Conductivity for Multifunctional Wearable Sensors. ACS Appl. Polym. Mater..

[223] Yang X., Duan X., Zhu L., Zhao X., Zhang S., Ni S., Wu S., Han Z., Deng W., Sun D. (2025). Carbon-dot-Zn^2+^ assembly hybrid multifunctional hydrogels for synergistically monitoring local glucose and repairing diabetic wound. ACS Appl. Mater. Interfaces.

[224] Cao Y., Cheng Y., Zhao G. (2021). Near-infrared light-, magneto-, and pH-responsive GO–Fe3O4/poly (N-isopropylacrylamide)/alginate nanocomposite hydrogel microcapsules for controlled drug release. Langmuir.

[225] Zhang L., Bei Z., Li T., Qian Z. (2023). An injectable conductive hydrogel with dual responsive release of rosmarinic acid improves cardiac function and promotes repair after myocardial infarction. Bioact. Mater..

[226] Li N., Wang X., Liu Y., Li Y., Li J., Qin Z., Jiao T. (2024). Ultrastretchable, Self-Adhesive and conductive MXene nanocomposite hydrogel for body-surface temperature distinguishing and electrophysiological signal monitoring. Chem. Eng. J..

[227] Wang J., Hou Z., Wang W., Bai L., Chen H., Yang L., Yin K., Yang H., Wei D. (2025). Design of self-healing nanocomposite hydrogels and the application to the detection of human exercise and ascorbic acid in sweat. Biosens. Bioelectron..

[228] Li W., Zou K., Guo J., Zhang C., Feng J., You J., Cheng G., Zhou Q., Kong M., Li G. (2024). Integrated fibrous iontronic pressure sensors with high sensitivity and reliability for human plantar pressure and gait analysis. ACS Nano.

[229] Tang J., Chen Y., Li K., Bai Y., Guo R., Fukuda K., Someya T., Ito Y., Kawamoto M. (2025). Ultra-low hysteresis, high-toughness, anti-swelling nanocomposite hydrogel derived from dynamic multiple noncovalent interactions for wearable strain sensing and underwater gait recognition. Chem. Eng. J..

[230] Ahmed I., El Turk S., Al Ghaferi A., Samad Y.A., Butt H. (2024). Nanocomposite Hydrogel-Based Optical Fiber Probe for Continuous Glucose Sensing. Small Sci..

[231] Guo J., Zhou B., Du Z., Yang C., Kong L., Xu L. (2021). Soft and plasmonic hydrogel optical probe for glucose monitoring. Nanophotonics.

[232] Zhang Y., Zhao G., Zheng M., Hu T., Yang C., Xu C. (2023). A nanometallic conductive composite-hydrogel core–shell microneedle skin patch for real-time monitoring of interstitial glucose levels. Nanoscale.

[233] GhavamiNejad P., GhavamiNejad A., Zheng H., Dhingra K., Samarikhalaj M., Poudineh M. (2023). A conductive hydrogel microneedle-based assay integrating PEDOT: PSS and Ag-Pt nanoparticles for real-time, enzyme-less, and electrochemical sensing of glucose. Adv. Healthc. Mater..

[234] Kim G.J., Kim K.O. (2020). Novel glucose-responsive of the transparent nanofiber hydrogel patches as a wearable biosensor via electrospinning. Sci. Rep..

[235] Eskilson O., Zattarin E., Berglund L., Oksman K., Hanna K., Rakar J., Sivlér P., Skog M., Rinklake I., Shamasha R. (2023). Nanocellulose composite wound dressings for real-time pH wound monitoring. Mater. Today Bio.

[236] Lei H., Yu X., Fan D. (2024). Nanocomposite hydrogel for real-time wound status monitoring and comprehensive treatment. Adv. Sci..

[237] Sheets K.M., Dominguez J.C., Keating C.J., Keleher J.J. (2025). Understanding the Bacterial-Nanocomposite Interfacial Dynamics of a Two-Layer Responsive Hydrogel Network with Wound Healing Applications. Electrochemical Society Meeting Abstracts 247.

[238] Duong T.K.N., Truong T.T., Phan T.N.L., Nguyen T.X., Doan V.H.M., Vo T.T., Choi J., Pal U., Dhar P., Lee B. (2025). Hydrogel-Based Smart Materials for Wound Healing and Sensing. Aggregate.

[239] Parshad B., Hu X., Nagle I., Rajput S.S., Peng F., Roussakis E., Cascales J.P., McPartland T., Wiatrowski A., Slade J.B. (2025). Hydrogel nanocomposite-based breathable non-adhesive coating for oxygen sensing film used within extracorporeal perfusion system. Sci. Rep..

[240] Jia L., Li Y., Ren A., Xiang T., Zhou S. (2024). Degradable and recyclable hydrogels for sustainable bioelectronics. ACS Appl. Mater. Interfaces.

[241] Hosseini E.S., Dervin S., Ganguly P., Dahiya R. (2020). Biodegradable materials for sustainable health monitoring devices. ACS Appl. Bio Mater..

[242] Lee D.H., Park T., Yoo H. (2022). Biodegradable polymer composites for electrophysiological signal sensing. Polymers.

[243] de Marzo G., Mastronardi V.M., Todaro M.T., Blasi L., Antonaci V., Algieri L., Scaraggi M., De Vittorio M. (2024). Sustainable electronic biomaterials for body-compliant devices: Challenges and perspectives for wearable bio-mechanical sensors and body energy harvesters. Nano Energy.

[244] Popescu M., Ungureanu C. (2023). Biosensors in food and healthcare industries: Bio-coatings based on biogenic nanoparticles and biopolymers. Coatings.

[245] Nath N., Chakroborty S., Vishwakarma D.P., Goga G., Yadav A.S., Mohan R. (2024). Recent advances in sustainable nature-based functional materials for biomedical sensor technologies. Environ. Sci. Pollut. Res..

[246] Gu J., Huang J., Chen G., Hou L., Zhang J., Zhang X., Yang X., Guan L., Jiang X., Liu H. (2020). Multifunctional poly (vinyl alcohol) nanocomposite organohydrogel for flexible strain and temperature sensor. ACS Appl. Mater. Interfaces.

[247] Long Y., Wang Z., Xu F., Jiang B., Xiao J., Yang J., Wang Z.L., Hu W. (2022). Mechanically Ultra-Robust, elastic, conductive, and Multifunctional hybrid Hydrogel for a triboelectric nanogenerator and flexible/wearable sensor. Small.

[248] Liang Q., Xia X., Sun X., Yu D., Huang X., Han G., Mugo S.M., Chen W., Zhang Q. (2022). Highly stretchable hydrogels as wearable and implantable sensors for recording physiological and brain neural signals. Adv. Sci..

[249] He Z., Yuan W. (2021). Highly stretchable, adhesive ionic liquid-containing nanocomposite hydrogel for self-powered multifunctional strain sensors with temperature tolerance. ACS Appl. Mater. Interfaces.

[250] He F., You X., Gong H., Yang Y., Bai T., Wang W., Guo W., Liu X., Ye M. (2020). Stretchable, biocompatible, and multifunctional silk fibroin-based hydrogels toward wearable strain/pressure sensors and triboelectric nanogenerators. ACS Appl. Mater. Interfaces.

[251] Liao H., Guo X., Wan P., Yu G. (2019). Conductive MXene nanocomposite organohydrogel for flexible, healable, low-temperature tolerant strain sensors. Adv. Funct. Mater..

[252] Zhao L., Ren Z., Liu X., Ling Q., Li Z., Gu H. (2021). A multifunctional, self-healing, self-adhesive, and conductive sodium alginate/poly (vinyl alcohol) composite hydrogel as a flexible strain sensor. ACS Appl. Mater. Interfaces.

